# FBXO11 suppression rewires an NPM1-centered interactome influencing the progression of myelodysplastic syndrome

**DOI:** 10.1172/JCI193636

**Published:** 2026-01-16

**Authors:** Madeline Niederkorn, Lavanya Bezavada, Anitria Cotton, Lance E. Palmer, Lahiri Konada, Trent Hall, Vishwajeeth R. Pagala, Jinbin Zhai, Zuo-Fei Yuan, Yingxue Fu, Jacob A. Steele, Shilpa Narina, Andrew Schild, Chengzhou Wu, Sarah Aminov, Michael Schieber, Erin McGovern, Aaron B. Taylor, Sandeep Gurbuxani, Peng Xu, Peng Ji, Laura J. Janke, Anthony A. High, Guolian Kang, Shondra M. Pruett-Miller, Mitchell Weiss, Amit Verma, Raajit K. Rampal, John D. Crispino

**Affiliations:** 1 Department of Hematology, Division of Experimental Hematology,; 2 Center for Proteomics and Metabolomics,; 3 Center for Advanced Genome Engineering and Department of Cell and Molecular Biology, and; 4 Department of Biostatistics, St. Jude Children’s Research Hospital, Memphis, Tennessee, USA.; 5 Department of Oncology, Montefiore Einstein Comprehensive Cancer Center, Bronx, New York, USA.; 6 Northwestern Medicine, Lake Forest Hospital, Lake Forest, Illinois, USA.; 7 Memorial Sloan Kettering Cancer Center, New York City, New York, USA.; 8 Cell and Tissue Imaging Center, St. Jude Children’s Research Hospital, Memphis, Tennessee, USA.; 9 Department of Pathology, University of Chicago, Chicago, Illinois, USA.; 10 Cyrus Tang Medical Institute, Suzhou Medical Collage, Soochow University, Suzhou, Jiangsu Province, China.; 11 Department of Pathology, Northwestern University, Chicago, Illinois, USA.; 12 Department of Pathology, Division of Comparative Pathology, St. Jude Children’s Research Hospital, Memphis, Tennessee, USA.

**Keywords:** Hematology, Oncology, Leukemias, Ubiquitin-proteosome system

## Abstract

Myelodysplastic syndromes (MDSs) are malignant hematopoietic stem and progenitor cell (HSPC) disorders that lead to ineffective blood production with poor outcomes. We previously showed that F-box only protein 11 (FBXO11) is downregulated in MDS, and here we report how this event contributes to disease progression. Integration of multiomics data revealed that the SCF-FBXO11 complex regulates spliceosome and ribosome components in a nucleophosmin 1 (NPM1)-centric network. FBXO11 facilitates the ubiquitylation of NPM1, whereby deletion of FBXO11 results in the reorganization of NPM1 and a de-repression of alternative splicing. Label-free total quantitative proteomics demonstrated that the FBXO11-NPM1 interactome was markedly downregulated in cells from patients with CD34^+^ MDS. In addition, we discovered that MYC was evicted from the *FBXO11* promoter by TLR2 activation, revealing that it was a MYC target gene and explaining why *FBXO11* expression was decreased in MDS. In MDS mouse models, genetic ablation of *Fbxo11* exacerbated neutropenia concomitant with a profound decrease in NPM1 protein levels. Finally, we discovered rare mutations in *FBXO11*, which mapped to a previously unstudied functional intrinsically disordered region (IDR) in the N-terminus responsible for binding NPM1. These data support a model in which FBXO11 rewires RNA binding and ribosomal subnetworks through ubiquitylation of NPM1, ultimately restricting MDS progression.

## Abstract 2

FBXO11 is mutated in myeloid malignancies and its deficiency alters NPM1 and the proteomic network of splicing regulators in MDS.

## Graphical abstract

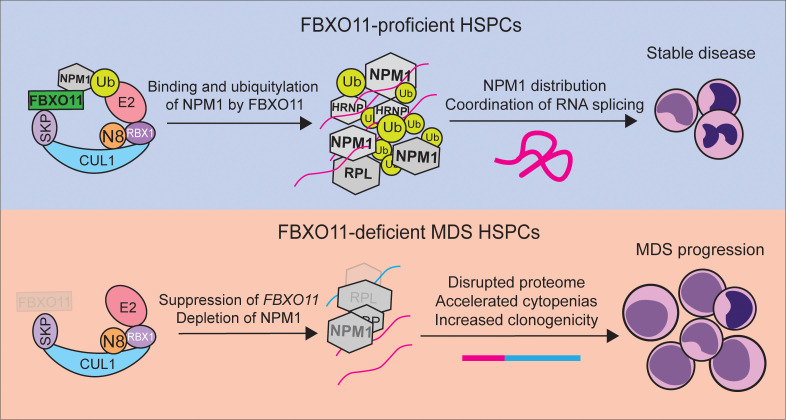

## Introduction

Myelodysplastic syndrome (MDS) affects 4 in 100,000 individuals in the United States, with over 13,000 new cases annually (1) and is caused by mutations that lead to ineffective blood production and poor outcomes (2–4). The majority of patients die of hemorrhage and unresolved cytopenias or from transformation to secondary acute myeloid leukemia (sAML) (3, 5). Survival rates for patients with MDS or sAML are approximately 37% and less than 30%, respectively (1, 6).

Depending on the molecular subtype of MDS and risk stratification of patients (7), treatment can include supportive approaches such as erythropoietin, luspatercept, or blood transfusions, which provide modest clinical benefit. Higher-risk patients with life-threatening cytopenias and poor prognostic cytogenetics receive the hypomethylating agents decitabine or azacitidine to prolong survival, often with little improvement in their overall quality of life (7, 8). The only curative therapy for MDS or resultant sAML is allogeneic stem cell transplantation (6), and this has recently been extended to include HLA-haploidentical donors (9). However, many patients are ineligible for these potentially curative approaches because of age-related comorbidities and health complications arising from the disease such as infection and bleeding. Furthermore, the success rates of recent clinical trials with the goal of extending patient survival have unfortunately been low (10), leaving more tractable curative approaches for MDS an unmet clinical need. This points to a gap in our understanding of the biology of MDS hematopoietic stem and progenitor cells (HSPCs) that underlies the lack of durable responses or curative potential with currently available therapies. Across hematologic malignancies, new therapies have been made possible by major advancements in understanding the genomics, cytogenetics, and gene expression programs in patients’ cells (11), increasingly so at the single-cell level. Of note, the MDS proteome remains unexplored.

We previously performed a whole-genome CRISPR-KO screen to identify genes that support MDS cell survival and identified the F-box protein 11 (FBXO11) as a leading candidate (12). *FBXO11* is mutated in lymphoid malignancies (13–15), required for normal hematopoiesis (16, 17), expressed at low levels in a subset of patients with AML (12), and implicated in regulating MHC class II expression in AML (18). F-box proteins such as FBXO11 are substrate receptors that determine the specificity for ubiquitylating enzyme complexes termed SKP1–CUL1–F-box (SCF) ligases (19). Ubiquitylation is an abundant posttranslational modification that was originally identified as a degradation signal (20). Decades of research have elucidated additional roles for ubiquitylation and its varied chain types in autophagy, innate-immune complex scaffolding, epigenetic histone modifications, and DNA damage responses, which have been summarized in several reviews (21–24). Many of these pathways are hallmarks of cancer development (25), which make the ubiquitylating enzyme complexes that regulate them attractive to mine for therapeutic vulnerabilities.

In our study, we aimed to define the role of FBXO11 downregulation in MDS progression by identifying disease-relevant substrates and evaluating its contribution to MDS pathogenesis. We postulate that a deeper understanding of the cellular mechanisms underlying MDS progression and the protein networks that dynamically rewire them will aid the discovery of new therapeutic strategies.

## Results

### Integrated proteomics defines nucleophosmin 1 as an SCF-FBXO11 substrate.

To determine the molecular interactions of FBXO11 relevant to MDS progression, we devised a multipronged approach to identify candidate substrates for ubiquitylation mediated by FBXO11. First, we reanalyzed ubiquitin proteomics data from WT versus FBXO11-deficient MDS-AML cells (12) to identify proteins that quantitatively lost ubiquitylation in the absence of FBXO11. Next, we performed co-IP and mass spectrometry (MS) analyses of FBXO11 in MDS-AML cells to detect proteins in FBXO11 complexes. Last, we used the Ub proteomics data to generate a focused sgRNA library and performed a targeted CRISPR/Cas9 screen to evaluate the effect of the loss of these proteins on MDS-AML colony-forming ability.

**Figure 1:**
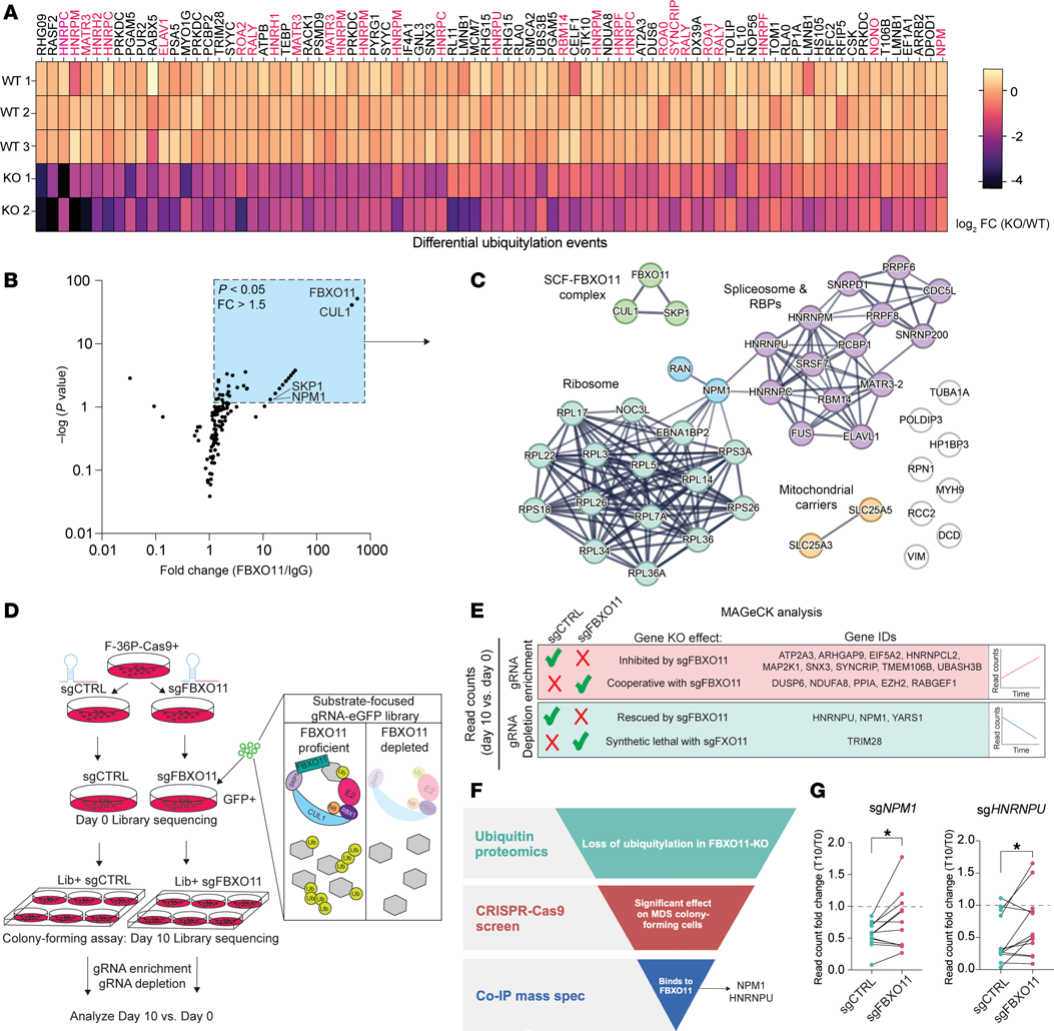
Integrated multiomics defines NPM1 as an SCF-FBXO11 substrate. (**A**) Heatmap of log_2_FC values for ubiquitylated peptides in MDS-L FBXO11-KO versus WT cells. Shown are hits with a FC of greater than |0.5|. Known RNA-binding proteins are labeled in red. Data were reanalyzed from ref. 12. (**B**) Scatter plot of the results from co-IP and MS identification of endogenous FBXO11 complexes in F-36P cell nuclear fractions. Shown are the –log(*P* value) against the FC enrichment of individual proteins identified in the FBXO11 IP versus the IgG control IP. The blue box highlights proteins with a *P* value of less than 0.05 and a FC of greater than 1.5. The *P* value was derived by G test. (**C**) STRING network analysis of FBXO11-interacting proteins identified by MS. Only input nodes are shown, with the threshold cutoff of medium confidence at 0.7. The thickness of the lines connecting nodes indicates the relative strength of evidence supporting the protein-protein interactions. (**D**) Strategy for the FBXO11 substrate-focused CRISPR/Cas9 screen, in which the gRNA library encompasses all of the differentially ubiquitylated peptides identified from the ubiquitin proteomics experiment represented in **A**. (**E**) Summary table of significant hits from the MAGeCK (Model-based Analysis of Genome-wide CRISPR-Cas9 Knockout) analysis of the CRISPR screen in **D**, demonstrating selective enrichment or depletion of guides in sgCTRL or sgFBXO11 colonies. (**F**) Schematic overview of the integrated multiomics approach to identify relevant candidate FBXO11 substrates meeting the listed criteria, revealing NPM1 and HNRNPU. (**G**) FC in individual gRNA read counts for NPM1 and HNRNPU in the colony-forming assay (day 10) versus initial representation (day 0) in the CRISPR screen. The FC for individual guides was compared between sgCTRL and sgFBXO11 experimental groups, shown on the graphs. *n* = 6 guides per gene, in 2 independent biological replicates of the CRISPR screen. *P < 0.05, by 2-tailed, paired *t* test.

In quantifying the ubiquitylated peptide intensities with a fold change (FC) of greater than|0.5| reported in the FBXO11-KO/WT MDS-L cells, we observed enrichment of RNA-binding proteins (Figure 1A, red). To clarify which substrates are more likely to be direct targets of the SCF-FBXO11 complex, we performed co-IP of FBXO11 complexes in F-36P cells (26), established from a patient with MDS-refractory anemia with excess blasts that had progressed to AML. FBXO11 is most abundantly expressed in the nucleus, so we performed nuclear fractionation followed by endogenous FBXO11 co-IP and liquid chromatography/MS (LC/MS) (Supplemental Figure 1A; supplemental material available online with this article; https://doi.org/10.1172/JCI193636DS1). We identified significant interactors with FBXO11, including, as expected, the SCF complex members S-phase kinase associated protein 1 and cullin 1 in high abundance (Figure 1B and Supplemental Table 1). Through STRING network analysis, we observed 2 functional nodes of highly interconnected proteins: the spliceosome and ribosomal subunits (Figure 1C and Supplemental Figure 1B).

To assess which of the ubiquitylated proteins contribute to MDS/AML clonogenicity, we conducted a CRISPR-KO assay of candidate substrates. We designed an sgRNA library to target all the differentially ubiquitylated proteins identified in FBXO11-KO MDS-L cells (Figure 1D). We then transduced Cas9-expressing F-36P cells with the target-focused sgRNA library and performed colony-forming assays as a readout of clonogenic potential. We performed this screen in F-36P control and FBXO11-depleted cells (Supplemental Figure 1C), allowing us to focus on the genes with whose KO effects in the clonogenic assay were dependent on FBXO11 expression (Figure 1E, Supplemental Figure 1D, and Supplemental Table 2). Integration of all 3 datasets identified nucleophosmin 1 (NPM1) and HNRNPU as the lead candidate substrates of FBXO11 (Figure 1, F and G). Both proteins lost ubiquitylation events when FBXO11 was deleted, showed FBXO11-dependent effects on MDS progenitor colony-forming ability by CRISPR-KO, and were detected in endogenous FBXO11 complexes in the F-36P cells. While *NPM1* is the most frequently mutated gene in de novo AML (27), it is rarely mutated in MDS or other chronic myeloid disorders (28). Studies in *Npm1*-deficient mice showed that loss of NPM1 contributes to MDS phenotypes including premature aging of hematopoietic stem cells (HSCs) (29) and a decrease in active HSCs (30). In patients with MDS, loss of NPM1 could occur in when common deletions on chromosome 5q include the *NPM1* locus (31). Indeed, NPM1 protein is downregulated in the majority of previously evaluated MDS and AML patient samples (29), irrespective of del(5q) status. Thus, we focused on NPM1 as a key candidate substrate for SCF-FBXO11 ubiquitylation that is relevant to the pathogenesis of MDS.

We observed biologically relevant hits in the CRISPR/Cas9-KO screen that did not meet our criteria for direct FBXO11 substrates. Read counts for EZH2 guides were enriched in FBXO11-depleted colonies (Supplemental Figure 1E). We previously reported that destabilizing EZH2 splicing was increased in samples from patients with MDS or secondary AML expressing low levels of FBXO11 (12). Moreover, loss of EZH2 is an independent prognostic indicator of worsened outcomes in patients with MDS (32). Our data suggest that dual loss of FBXO11 and EZH2 may accelerate disease progression. Additionally, guides targeting the RNA-binding protein SYNCRIP were enriched only in the single-guide control (sgCTRL) colonies (Supplemental Figure 1E). SYNCRIP has been studied in the context of AML, where its overexpression supported leukemic stem cell self-renewal and normal hematopoietic stem cells (33, 34). Interestingly, we found that our sgRNA sequences exclusively targeted a SYNCRIP sequence unique to its nuclear isoform (Supplemental Figure 1F), suggesting that loss of this SYNCRIP isoform may be important for MDS, distinct from the canonical isoform studied in AML. By multiplexing FBXO11 and SYCNRIP gRNAs, we observed partial rescue of the colony-forming ability imbued by sgFBXO11 in vitro (Supplemental Figure 1G). FBXO11-deficient cell states may cooperate with the loss of EZH2 or SYNCRIP during MDS progression and would be worth exploring in future studies.

### FBXO11 facilitates ubiquitylation of NPM1 and its distribution into the nucleoplasm.

**Figure 2:**
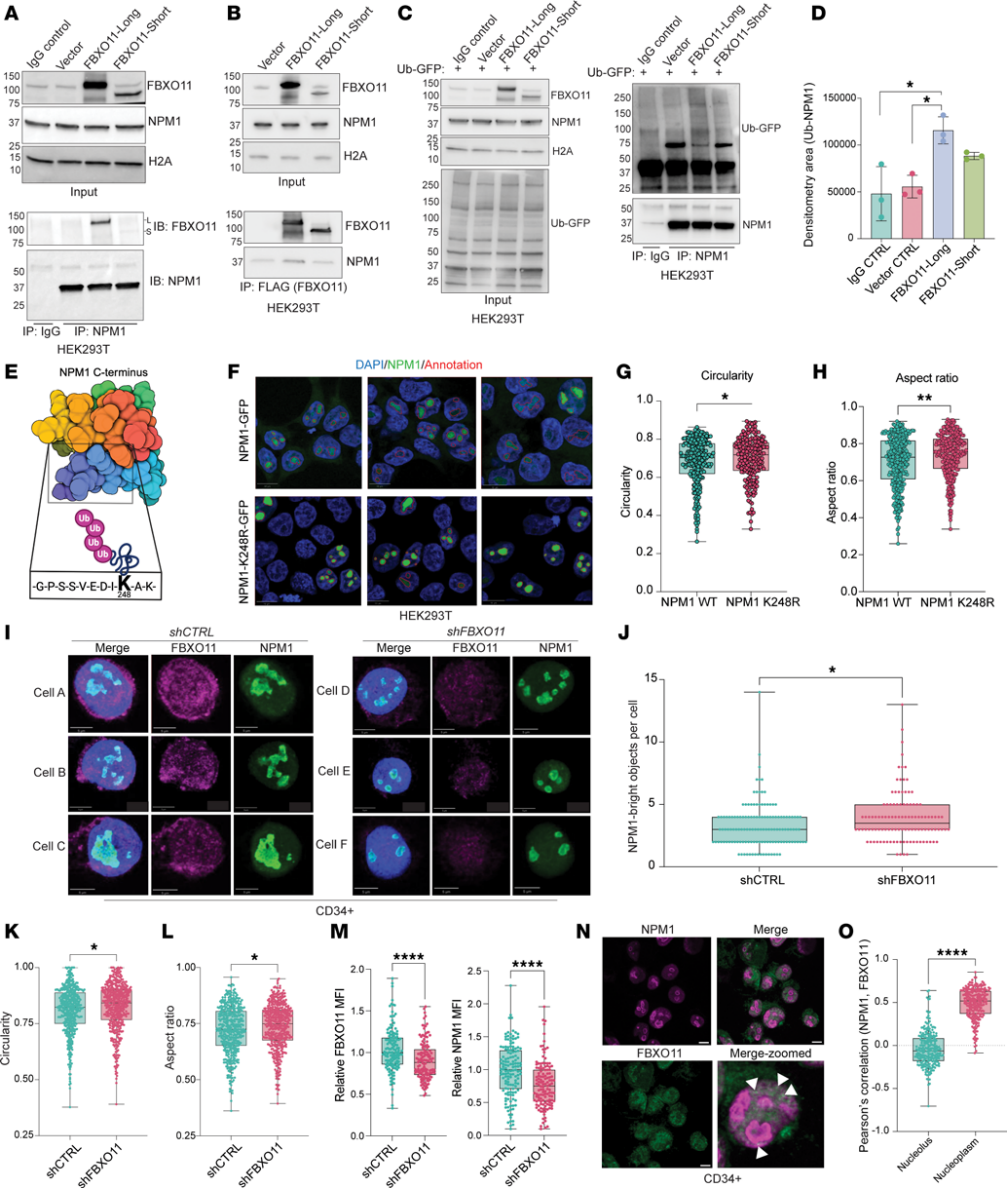
FBXO11 facilitates ubiquitylation of NPM1 and permits its distribution into the nucleoplasm. (**A**) Immunoblots of overexpressed FLAG-FBXO11 isoforms in HEK293T cells. Blotting was done for endogenous NPM1 and H2A. Endogenous NPM1 IPs were probed for FBXO11 and NPM1. (**B**) Immunoblots of co-IP FLAG-FBXO11 complexes of FBXO11 and NPM1. (**C**) Left: Input immunoblots for FLAG-FBXO11, NPM1, ubiquitin-GFP, and H2A in HEK293T cells. IPs of NPM1 were performed to detect ubiquitylation by FBXO11. Right: NPM1 versus IgG control IP, immunoblotted for GFP-ubiquitin. *n* = 3. (**D**) Densitometry for NPM1 poly-ubiquitin bands from **C**. The area quantified is 75 kDa and above. *n* = 3. *Q < 0.05, by Kruskal-Wallis ANOVA corrected for multiple comparisons by the Benjamini method. (**E**) Schematic depicting K248-Ub of NPM1 in its C-terminal core. The ubiquitin (Ub) footprint was identified by MS, Figure 1A and Supplemental Table 1. (**F**) HEK239T cells were transfected with NPM1-GFP or NPM1-K248R-GFP fusions. Red outlines indicate GFP-bright regions annotated in QuPath. Scale bars: 10 μm. (**G** and **H**) Annotated regions quantified for circularity (**G**) and aspect ratio (**H**). *n* = 2. Dots represent individual NPM1^+^ regions.*P < 0.05 and **P < 0.01, by 2-tailed Mann-Whitney *U* test. (**I**) Confocal images of human CD34^+^ cells expressing sh*CTRL* or sh*FBXO11* mCherry constructs. Images are pseudocolored for FBXO11 (magenta), NPM1 (green), and DAPI (blue). Scale bars: 5 μm. (**J**) NPM1-bright objects per cell. *P < 0.05, by 2-tailed Mann-Whitney *U* test. (**K**) Circularity values for NPM1-bright objects in the shCTRL and shFBXO11 images. *P < 0.05, by 2-tailed Mann-Whitney *U* test. (**L**) Aspect ratio values for NPM1-bright objects in the shCTRL and shFBXO11 images. *P < 0.05, by 2-tailed Mann-Whitney *U* test. (**M**) Relative MFI values for FBXO11 signal and NPM1 signal on a per-cell basis. ****P < 0.0001, by 2-tailed *t* test. (**N**) Human CD34^+^ cells stained for FBXO11 (green) and NPM1 (magenta). Colocalization (white arrowheads) where green and magenta overlap. Scale bars: 5 μm. (**O**) Pearson correlation values for NPM1 and FBXO11 signals in the nucleolus (NPM1-bright) and nucleoplasm (NPM1-dim). Each dot represents 1 cell, with greater than 100 cells at ×63. ****P < 0.0001, by 2-tailed Mann-Whitney *U* test.

We validated the interaction of FBXO11 with NPM1 in the nucleus cells by reciprocal co-IP assays in HEK293T cells. We exogenously expressed the 2 major isoforms of FBXO11: the canonical *FBXO11* transcript variant 4 (FBXO11-long) or the *FBXO11* transcript variant 1 (FBXO11-short), which utilizes an alternative start site that skips the first 84 amino acids. FBXO11-long showed enhanced binding to NPM1 in reciprocal co-IPs (Figure 2, A and B). We expected that ubiquitylation of NPM1 would be increased through this interaction, so we performed a ubiquitylation assay, in which we overexpressed FBXO11-long or FBXO11-short with GFP-tagged ubiquitin and precipitated NPM1 (Figure 2C). We observed a significant increase in ubiquitylated NPM1 with the expression of FBXO11-long, but not FBXO11-short (Figure 2D), consistent with our observations that FBXO11-short did not efficiently interact with NPM1. Our data demonstrate that interaction with FBXO11 was sufficient to induce polyubiquitylation of NPM1.

To map the ubiquitylation site of NPM1, we analyzed the Ub proteomics dataset and identified the di-glycine (GG) ubiquitin footprint on lysine (K) 248 of NPM1. The K248 ubiquitylated peptide was decreased in the FBXO11-KO MDS-L cells compared with control MDS-L cells, suggesting that this Ub event was mediated by FBXO11. K248 was located within the C-terminus of NPM1 (Figure 2E). The folded C-terminus is 1 monomer of the pentameric core of NPM1 in the nucleolus. Since overexpression of FBXO11 did not degrade NPM1, we hypothesized that ubiquitylation of NPM1 would affect its nuclear distribution, dynamically affecting its molecular function (35). We generated NPM1-GFP fusion expression constructs and mutated K248 to an arginine to prevent its ubiquitylation by the SCF-FBXO11 complex. We expressed NPM1-GFP and NPM1-K248R-GFP in HEK293T cells and compared NPM1 expression and localization (Figure 2F). Strikingly, the K248R mutant formed rounded, more restricted clusters in the nucleoli of the cells compared with WT NPM1, which showed diffusivity (Figure 2F). To quantify these patterns, we measured the circularity and aspect ratio, which are calculated descriptors of shape elongation (Figure 2, G and H).

We next evaluated the effects of FBXO11 on NPM1 distribution in primary human HSPCs (Figure 2I). When we knocked down *FBXO11* in CD34^+^ cells using shRNA, we also observed that NPM1 formed more frequent and less elongated clusters in the nucleus (Figure 2, J–L), consistent with our observations using the K248R mutant in HEK293T cells. While NPM1 formed more frequent clusters in shFBXO11 cells, the normalized MFI of total NPM1 was significantly decreased (Figure 2M). This suggests that ubiquitylation of NPM1 by SCF-FBXO11 ultimately protected NPM1 protein levels in the nucleus of HSPCs. We observed a significantly stronger correlation of NPM1 and FBXO11 in the nucleoplasm of CD34^+^ cells compared with the nucleolar subcompartment (Figure 2, N and O).

### Low FBXO11 expression leads to de-repression of splicing through NPM1 and is associated with alternative splicing events in MDS.

**Figure 3:**
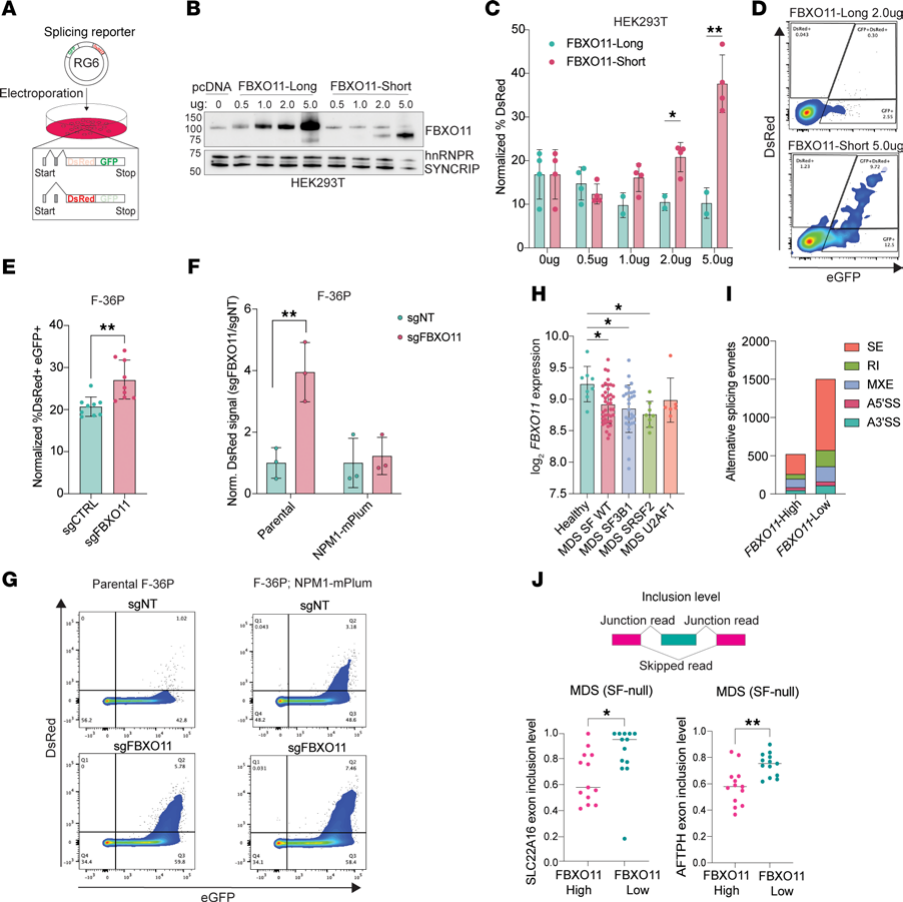
Low FBXO11 leads to derepression of splicing by NPM1 and is associated with alternative splicing events in MDS. (**A**) Schematic of splicing reporter delivered to F-36P cells. (**B**) Immunoblot for FBXO11 in HEK293T lysates with an increasing transfected plasmid dose of FBXO11-long and FBXO11-short, compared with endogenous hnRNPR and SYNCRIP. (**C**) Percentage of DsRed^+^ cells normalized to reporter-positive cells in **B** measured by FACS at 48 hours. *Q < 0.05 and **Q < 0.01, by multiple 2-tailed *t* tests corrected for multiple comparisons. (**D**) Representative flow plots of FBXO11-long and FBXO11-short reporter-positive cells. (**E**) DsRed-eGFP^+^ F-36P cell percentages normalized to reporter-positive cells. *n* = 3 independent experiments; *n* = 3 wells per group. **P < 0.01, by 2-tailed *t* test. (**F**) FC in DsRed^+^ cells in sgFBXO11 versus sgNT, in the context of parental F-36P cells or F-36P cells with NPM1 overexpression. **Q < 0.01. (**G**) Representative flow plots of sgFBXO1 versus sgNT in F-36P cells overexpressing NPM1. (**H**) log_2_FC of *FBXO11* expression in healthy control cells or MDS CD34^+^ samples, grouped by common splicing factor mutations. SF WT, splicing factor WT. *Q < 0.05, by 1-way ordinary ANOVA corrected for multiple comparisons using the Benjamini method. (**I**) Number of significant alternative splicing events determined by rMATS analysis. (**J**) Top: Schematic of junction reads versus skipped reads, used to determine exon inclusion levels for splicing events. Bottom: Exon inclusion levels for putative FBXO11-dependent splicing events in SLC22A16 and AFTPH in MDS CD34^+^ samples that were null for any splicing factor mutation. Each dot represents 1 patient.

Alternative splicing is a putative driver of MDS pathogenesis, and over 50% of patients with MDS harbor mutations in splicing factor mutations. Given that the FBXO11 interactome is composed of core spliceosome components and includes NPM1, on the basis of our data, we hypothesized that the FBXO11-NPM1 interaction could facilitate alternative splicing. To test this, we used a cassette exon splicing reporter system in vitro (36). We transfected increasing amounts of FBXO11-long or FBXO11-short in combination with the RG6 reporter plasmid into HEK293T cells. Skipping of the reporter cassette exon encodes DsRed, while inclusion of the exon, by default, encodes GFP (Figure 3A). Increased expression of FBXO11-long, which bound NPM1 in co-IPs, led to suppression of the in vitro splicing reporter (as the normalized percentage of DsRed). Conversely, FBXO11-short alleviated this suppression and demonstrated a greater proportion of exon skipping (Figure 3, B–D). We also performed the splicing assay on sgCTRL and sgFBXO11 F-36P cells and evaluated reporter expression at 24 hours (Figure 3E). In the sgFBXO11 group, we observed de-repression of cassette splicing, similar to that of FBXO11-short, which failed to interact with NPM1 (Figure 2, A and B). To test the contribution of NPM1 to splicing suppression, we generated subclones of F-36P cells that overexpressed an NPM1-mPlum fusion and then delivered control or FBXO11-targeted gRNAs. We hypothesized that NPM1-overexpressing cells would be less sensitive to the reporter de-repression observed when FBXO11 was knocked out in isolation. In the context of NPM1 overexpression, sgFBXO11 failed to drive a significant change in the DsRed signal relative to the sgNT controls (Figure 3, F and G).

To evaluate FBXO11-mediated splicing in patients’ samples, we analyzed RNA-Seq data from MDS CD34^+^ cells (37). We observed significantly lower expression of *FBXO11* compared with expression in healthy controls, irrespective of the most common splicing factor mutations (Figure 3H). We also found that low *FBXO11* expression in MDS (third quartile) was associated with a greater number of disease-associated alternative splicing events than was seen in samples with high *FBXO11* expression (first quartile) compared with healthy controls (Figure 3I). There were more skipped exon events in *FBXO11*-low samples. We then compared the alternative splicing events in *FBXO11*-low MDS versus healthy samples and focused on events in MDS samples that lacked any splicing factor mutations (Supplemental Figure 2A). We observed significant, aberrant exon inclusion in putative *FBXO11*-associated events in the *SLC22A16* and *AFTPH* genes (Figure 3J). Furthermore, we validated the specific exon inclusion events in HUDEP2 erythroid progenitor cells with KO of *FBXO11*, which were previously sequenced (Supplemental Figure 2, B and C) (17, 37). These findings support a model in which, through its ubiquitin substrate receptor function, FBXO11 rewired an NPM1-centric RNA-binding protein network that suppressed SE splicing events in MDS.

### The FBXO11-NPM1 nexus is perturbed in the MDS HSPC proteome.

**Figure 4:**
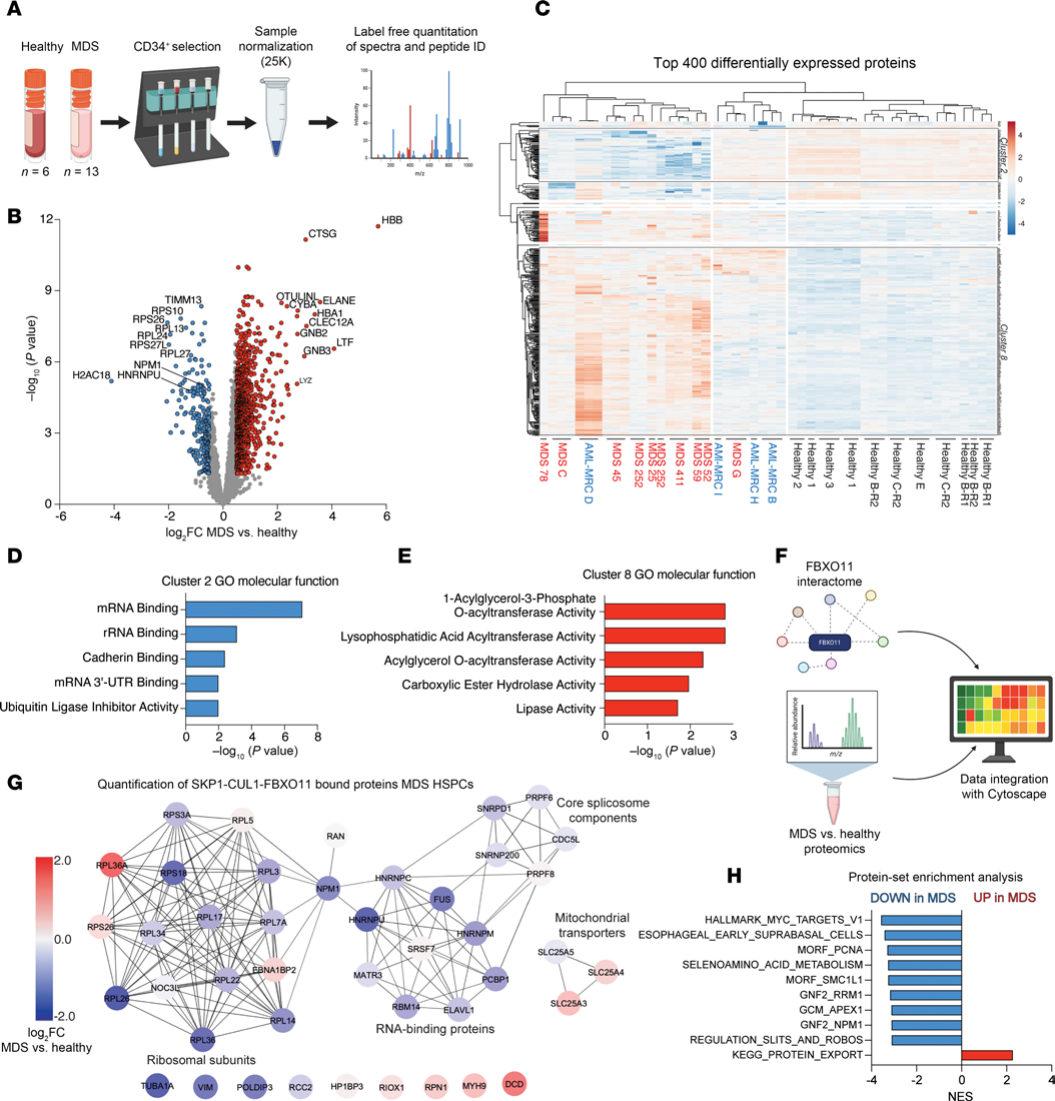
The FBXO11-NPM1 nexus is perturbed in the MDS HSPC proteome. (**A**) Workflow to isolate CD34^+^ cell fractions from healthy donor (*n* = 6) and MDS patient (*n* = 13) samples, to normalize to 25,000 live cells per replicate tube, and perform total quantitative proteomics by DIA of peptide spectra. Illustration was created in BioRender.com. (**B**) Volcano plot of differentially detected proteins in MDS samples versus healthy controls. Each data point indicates 1 protein, with coordinates reflecting the –log_10_ of the *P* value against its log_2_FC for patients with MDS (*n* = 13) versus healthy donors (*n* = 6). Color-coding indicates a *P* value cutoff of less than 0.05 and a log_2_FC cutoff >|0.5|. (**C**) Heatmap of the top 400 differentially expressed proteins in MDS versus healthy controls. Unsupervised hierarchical clustering of patient samples was performed using ClustVis. Each column represents 1 replicate tube from the indicated patient samples below the map. R1, run 1; R2, run 2. (**D**) GO molecular function analysis of cluster 2 proteins, the largest cluster downregulated in MDS. (**E**) GO molecular function analysis of cluster 8 proteins, the largest cluster upregulated in MDS. (**F**) Schematic depicting the data integration performed using Cytoscape to quantify the FBXO11 interactome in primary MDS HSPCs. Schematic was created in BioRender.com. (**G**) Resultant data visualization of the integrated proteomics analysis performed in **F**. Blue indicates down in MDS; red indicates up in MDS. (**H**) Normalized enrichment scores from GSEA of the significantly differentially expressed proteins in the MDS proteome versus healthy controls.

Using in vitro models, we established the ubiquitylation of NPM1 facilitated by FBXO11, providing the basis for how loss of FBXO11 could support MDS progression through disrupted splicing activity and RNA-binding protein (RBP) rewiring. To uncover which protein-protein interactions are altered in patient HSPCs, we applied a low-input proteomics pipeline and utilized data integration methods to assay the FBXO11-NPM1 network in MDS CD34^+^ cells. We performed total quantitative label-free MS of primary MDS CD34^+^ cells (*n* = 13 patients) and healthy control CD34^+^ cells (*n* = 6 donors) (Figure 4A and Supplemental Table 3). Principal component analysis using the top 50% most variable proteins we identified revealed that healthy CD34^+^ cells clustered away from MDS samples (Supplemental Figure 3A). Notably, expression levels of both NPM1 and HNRNPU protein were markedly lower in MDS HSPCs compared with healthy controls (Figure 4B) as well as several ribosomal subunits that are also part of the FBXO11 interactome. We integrated FC values of our MDS HSPC proteome data with reported FC values of the MDS HSPC transcriptome for all individual genes in both datasets and observed no correlation (Supplemental Figure 3B). Many of the downregulated RBPs and ribosomal subunits of the FBXO11 network showed relatively minor changes at the transcript level (|log_2_FC|<0.2), suggesting that posttranslational mechanisms like ubiquitylation imparted by the SCF-FBXO11 complex dominate control of the MDS proteome.

To dissect the MDS HSPC proteome, we performed unsupervised hierarchical clustering of the samples (38) (Figure 4C). All of the healthy controls clustered distinctly from MDS, whereas the MDS samples generated 2 separate clusters: one was predominantly composed of MDS samples (red), while the other contained samples from patients who were diagnosed with AML–myelodysplastic-related changes (MRCs) (blue, Supplemental Table 4). To classify the dominant molecular pathways in the MDS HSPC proteome, we performed gene ontology (GO) molecular function analysis for the largest clusters that were downregulated (cluster 2) or upregulated (cluster 8) relative to healthy controls (Figure 4, D and E). mRNA and rRNA binding proteins encompassed most downregulated proteins, whereas acylglycerol acyltransferases were strikingly represented in upregulated proteins in MDS samples.

To determine the extent to which the FBXO11-NPM1 protein-protein interaction network is disrupted in primary MDS, we integrated our FBXO11 interactome data generated in the STRING database with the quantitative proteomics data from the MDS CD34^+^ cells (Figure 4F). In support of our hypothesis that the FBXO11-NPM1 interactome is perturbed in MDS, we detected a broad loss of this network in the MDS samples compared with control samples, as represented by negative log_2_FC values from MDS samples versus healthy samples projected onto the FBXO11 interaction network (Figure 4G, –log2FC = blue; + log_2_FC = red). Gene set enrichment analysis (GSEA) of the FBXO11 interactome showed significant disruption of this network relative to the rest of the MDS HSPC proteome (Supplemental Figure 3C, normalized enrichment score [NES] = –3.11; *P* < 0.0001). Evaluating all gene sets in the MDS HSPC proteome showed that MYC targets were downregulated (Figure 4H). Other downregulated pathways included NPM1 and ribosome and spliceosome biology (Figure 4H and Supplemental Table 5). Our data, therefore, uncover substantial perturbation of the FBXO11-NPM1 nexus in MDS and, to our knowledge, provide the first dataset demonstrating that the HSPC proteome is remodeled during MDS pathogenesis and progression.

### MYC binds to the FBXO11 promoter, and its occupancy is diminished by TLR2 activation.

**Figure 5:**
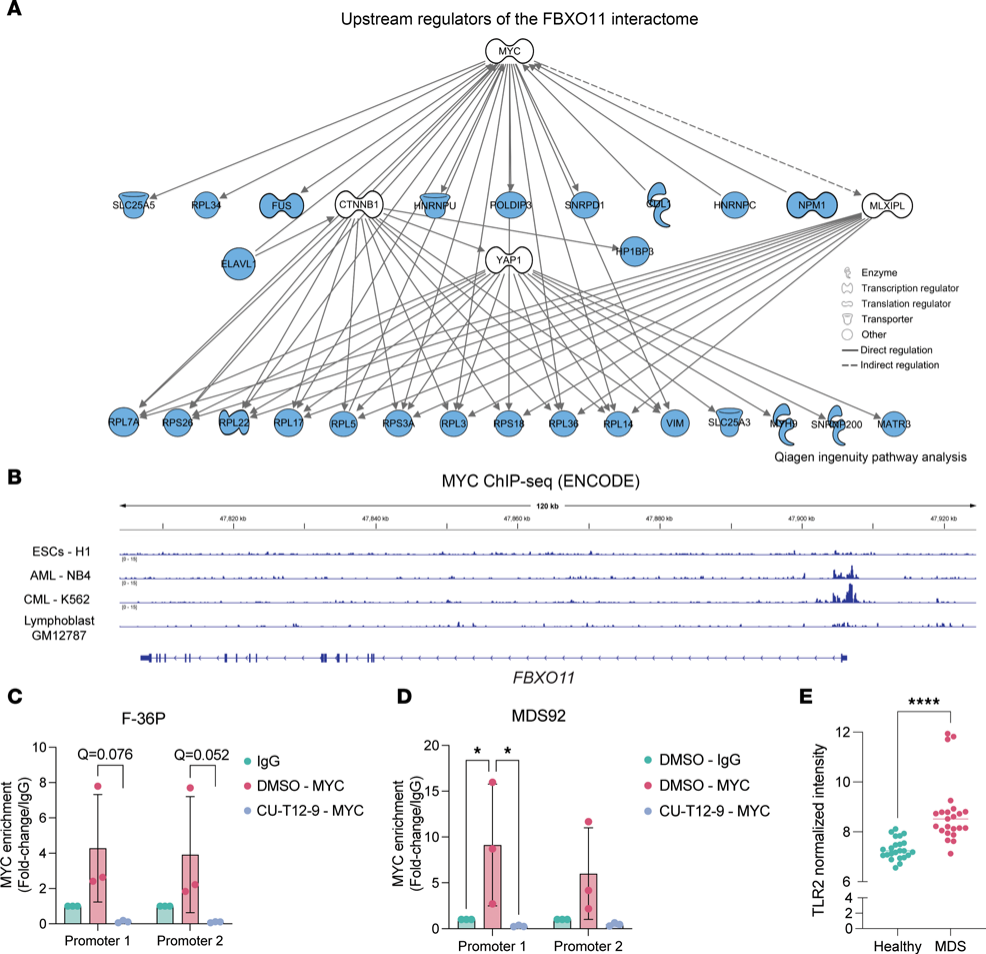
MYC binds the FBXO11 promoter, and this is suppressed by TLR2 activation in MDS. (**A**) Upstream regulator analysis of FBXO11-interacting proteins performed using IPA software. MYC was identified as a significant upstream regulator of the FBXO11 interactome (*P* = 1.07 × 10^15^). Shown is the MYC mechanistic network in hierarchical format, with FBXO11-interacting proteins in blue. (**B**) Integrative genomics viewer gene track of FBXO11 from MYC ChIP-Seq results in ENCODE. (**C** and **D**) FC of MYC binding to FBXO11 promoter sequences, calculated from MYC ChIP-qPCR in F-36P and MDS92 cells. TLR2 agonist (CU-T12-9) treatment resulted in a complete loss of MYC expression. *Q < 0.05, by 2-way ANOVA corrected for multiple comparisons using the Benjamini method. (**E**) TLR2 peptide intensity determined by quantitative proteomics from MDS patient sample replicates in which TLR2 peptides were detected. Each dot indicates 1 replicate. ****P < 0.0001, by 2-tailed Mann Whitney *U* test.

While other FBXO11-interacting proteins are predominantly affected at the protein level, FBXO11 itself is downregulated at the transcriptional level in MDS CD34^+^ cells (Figure 3H and Supplemental Figure 3B). In addition to the GSEA data (Figure 4H), Ingenuity Pathway Analysis (IPA) (39) inferred MYC as the upstream regulator of the FBXO11 subnetwork, at the top of the molecular mechanism hierarchy (Figure 5A). We hypothesized that *FBXO11* may be a direct target gene of MYC and that decreased MYC activity could explain how FBXO11 expression is diminished. To test this, we queried ChIP-Seq datasets in the ENCODE database to determine whether MYC binds the *FBXO11* promoter. In myeloid leukemia cell lines, we found binding of MYC at the *FBXO11* promoter but did not observe peaks in undifferentiated embryonic stem cell or lymphoblast lines (Figure 5B). To validate these observations in MDS cells, MYC occupancy at 2 regions in the FBXO11 promoter was assessed in F-36P and MDS92 cells. We found that MYC occupancy was enriched at the FBXO11 promoters compared with the IgG control sample (Figure 5, C and D). Moreover, since prior reports showed that TLR2-TRAF6 activation suppresses oncogenic MYC activity in myeloid malignancies (40), we tested whether the TLR2 agonist CU-T12-9 reduces MYC’s association with the *FBXO11* promoter. Indeed, we found that 24-hour treatment of F-36P and MDS92 cells with CU-T12-9 strongly blocked MYC binding to the *FBXO11* promoter sequences in the ChIP-qPCR assay (Figure 5, C and D). Importantly, our data also show that TLR2 itself was significantly upregulated in the MDS HSPC proteome (Figure 5E). This observation is in agreement with prior models, which showed that aberrant innate immune activation through TLRs — as part of a highly complex inflammatory milieu — impaired normal HSPC function and supported the expansion of MDS HSPCs (41). Our data support the model in which TLR2 activation in MDS blocked MYC-driven activation of *FBXO11*. Our studies demonstrate that the resultant loss of FBXO11 portended marked dysregulation of NPM1.

### FBXO11 deficiency enhances MDS HSPC fitness in vitro.

**Figure 6:**
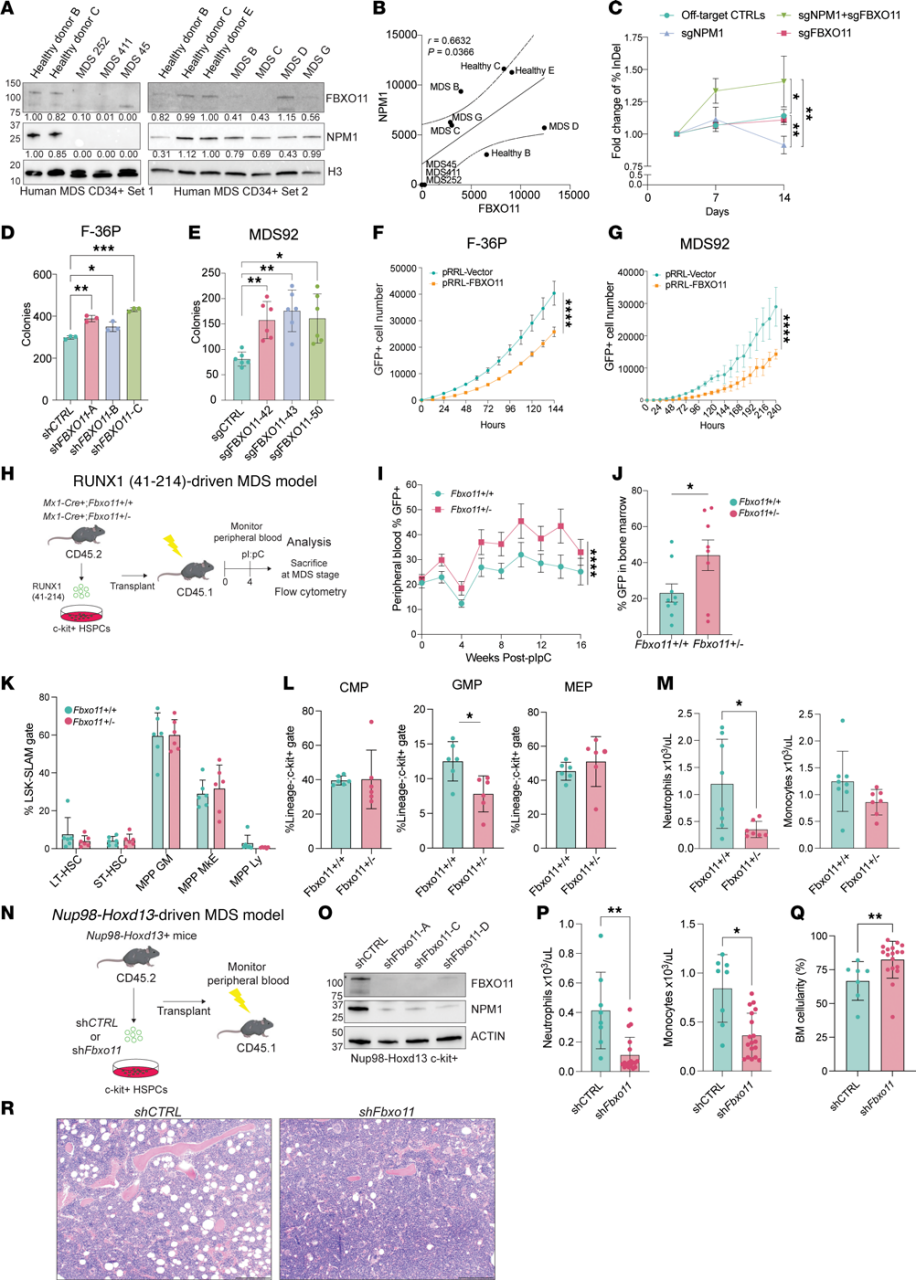
Genetic deletion of *Fbxo11* expands MDS progenitors in vitro and worsens murine MDS in vivo. (**A**) Immunoblots of FBXO11, NPM1, and H3 with densitometry. Each sample was normalized to H3, and then to controls. (**B**) Densitometric values of FBXO11 and NPM1 from **A**. Pearson correlation for all pairs of values and line of best fit with a 95% CI. (**C**) Cell fitness assay using primary CD34^+^ cells. FC in indel percentages at the end of the assay versus the initial read. For double knockout, the indel percentage tracks NPM1 edits. *Q < 0.05 and **Q < 0.01, by 2-group specific longitudinal mixed-effects analysis corrected for multiple comparisons. (**D**) Number of colonies. *Q < 0.05, **Q < 0.01, and ***Q < 0.001, by multiple *t* tests corrected for multiple comparisons. *n* = 3. (**E**) Number of colonies. *n* = 62 with 3 wells per assay. *Q < 0.05 and **Q < 0.01, by *t* tests on pooled replicates corrected for multiple comparisons. (**F**) Growth curve of F-36P GFP^+^ vector or FBXO11-overexpressing cells. *n* = 9 wells per group. ****P < 0.0001, by *t* test from the linear mixed-effects model with wells as a random effect. (**G**) Growth curve of MDS92 GFP^+^ vector or FBXO11-overexpressing cells. *n* = 9 wells per group. ****P < 0.0001, by *t* test from the linear mixed-effects model with wells as a random effect. (**H**) Schematic of the RUNX1-driven MDS mouse model on an inducible Mx1-Cre^+^ *Fbxo11^+/+^* or *Fbxo11^+/–^* background. (**I**) Percentage of GFP^+^ peripheral blood cells isolated from *Fbxo11^+/+^* RUNX1-GFP or *Fbxo11^+/–^* RUNX1-GFP transplants. *n* = 7–10 mice per group. ****P < 0.0001, by fixed-effects (type III) analysis. (**J**) Percentage of GFP^+^ mononuclear cells isolated from BM aspirates of transplant recipients at 11 weeks. *n* = 8–9 mice per group. *P-linear < 0.05, by 2-tailed *t* test. (**K**) Percentage contribution to the LSK, Lin^-^/Sca1^+^/Kit^+^/SLAM^+^ (signaling lymphocyte activation molecules) (LSK-SLAM^+^) gate of immunophenotypically defined HSPCs in surviving RUNX1 transplant recipients. *n* = 6 mice per group. LT-HSC, long-term HSC; ST-HSC, short-term HSC; MPP-GM, granulocyte-monocyte biased multipotent progenitors; MPP-MkE, megakaryocyte-erythroid biased multipotent progenitors; MPP Ly, lymphoid-biased multipotent progenitor. *Q* > 0.05, by *t* tests corrected for multiple comparisons (no significant differences were detected). (**L**) Percentage contribution of RUNX1-GFP^+^ common myeloid progenitor (CMP), GMP, and megakaryocyte-erythrocyte progenitor (MEPs) in the Lin^–^c-kit^+^ HSPC compartments. *n* = 6 mice per group. *P < 0.05, by multiple unpaired tests, *t* test for groups with normal distribution, or Mann-Whitney *U* test. (**M**) Differential CBCs in mice 16 weeks after pIpC. *P < 0.05, by unpaired, 2-tailed *t* for groups with normal distribution or Mann-Whitney *U* test. (**N**) Strategy of *Nup98-Hoxd13*-driven MDS mouse model with sh*CTRL* or sh*Fbxo11* vectors. *n* = 6–10 mice per group. (**O**) Western blot for FBXO11, NPM1, and ACTIN in c-kit^+^ cells from *Nup98-Hoxd13^+^* mice, transduced with lentiviral sh*CTRL* or sh*Fbxo11*. (**P**) CBC in *Nup98-Hoxd13* transplants at 2 months. sh*Fbxo11* groups were pooled. *P < 0.05 and **P < 0.005, by unpaired, 2-tailed *t* test for groups with normal distribution or Mann-Whitney *U* test. (**Q**) BM cellularity of *Nup98-Hoxd13* mice. sh*Fbxo11* groups were pooled. *P < 0.05 and **P < 0.01, by Mann-Whitney *U* test. (**R**) Representative H&E-stained femur cells from **Q**. Original magnification, ×10. Scale bar: 200 μm.

Next, we evaluated FBXO11 levels in primary MDS CD34^+^ cells and found that they was strikingly reduced in MDS versus CD34^+^ cells from healthy individuals along with a concomitant decrease in NPM1 expression (Figure 6A). We noted a positive correlation between FBXO11 and NPM1 protein expression levels (Figure 6B). We then tested whether the combined loss of FBXO11 and NPM1 could confer an advantage to HSPCs, as observed in MDS. We performed CRISPR-based cell fitness assays (42) to edit *NPM1* and *FBXO11* alone or in combination in human CD34^+^ HSPCs. On day 3 after electroporation, the initial edited pool was measured as the percentage of reads with insertion-deletion (indel) events in *NPM1* and *FBXO11* and monitored over time. By day 14, the relative proportion of NPM1-FBXO11 dual-edited cells in the pool had substantially expanded compared with single-gene edits (Figure 6C).

To evaluate the function of FBXO11 in human MDS cells, we used CRISPR/Cas9 or shRNA approaches to knock down FBXO11 in F-36P and MDS92 cells and assessed the effect on myeloid colony-forming ability of the cells in vitro (Figure 6, D and E, and Supplemental Figure 4A). We observed that reducing *FBXO11* increased the colony-forming ability of MDS cells, indicative of increased clonogenicity. To determine whether this effect was MDS disease specific, we knocked out *FBXO11* in healthy CD34^+^ HSPCs from 2 independent donors. A partial reduction in *FBXO11* expression in normal human HSPCs (Supplemental Figure 4B) led to a decrease in the colony-forming ability of granulocyte-monocyte progenitors (CFU-GMs), indicating that FBXO11 is important for normal myeloid colony formation. The number of erythroid colonies was unchanged (Supplemental Figure 4C). Similarly in normal mouse c-kit^+^ HSPCs, homozygous deletion of *Fbxo11* ex vivo impaired myeloid colony formation, with a trend toward decreased erythroid or multipotent progenitor colonies (Supplemental Figure 4D). To further test its role as a tumor suppressor in MDS, we overexpressed FBXO11 in F-36P and MDS92 cells. Overexpression of FBXO11 in both settings resulted in slower growth of the FBXO11-expressing GFP^+^ cells (Figure 6, F and G), concomitant with a trend toward increased apoptosis in F-36P cells, as determined by flow cytometry for annexin-V and DAPI (Supplemental Figure 4, E and F). Our data indicate that lower levels of FBXO11 uniquely supported MDS clonogenic potential, while it was detrimental to normal human and mouse myeloid progenitors in vitro.

### Knockdown of Fbxo11 exacerbates neutropenia in murine models of MDS.

On the basis of our in vitro findings, we hypothesized that depletion of *Fbxo11* by genetic approaches might accelerate MDS. We used a model of murine MDS using the retroviral transduction transplantation model of RUNX1 (41-214), which induces features of MDS within 4–6 months and a low incidence of AML in a subset of mice (43). To study the effects of FBXO11 on RUNX1-driven MDS, we isolated c-kit^+^ HSPCs from *Fbxo11^+/+^* *Mx1-Cre^+^* and *Fbxo11^+/fl^* *Mx1-Cre^+^* donor mice (CD45.2^+^) and expressed the human ortholog mutant RUNX1 (41-214) by retroviral transduction (Figure 6H). We normalized the number of RUNX1 (41-214) GFP^+^ cells between experimental groups and injected them into irradiated congenic recipient mice (CD45.1^+^). After 4 weeks of engraftment, both group of mice received injections of polyinosinic:polycytidylic acid (pIpC), which led to deletion of the single floxed allele of *Fbxo11* in the *Fbxo11^+/fl^* *Mx1-Cre^+^* group. We monitored peripheral blood counts and the percentage of RUNX1-GFP^+^ cells in the periphery (Supplemental Figure 4G). *Fbxo11^+/–^* recipient mice showed greater expansion of RUNX1 (41-214) GFP^+^ cells in the peripheral blood and bone marrow (BM) compared with controls (Figure 6, I and J). Although we did not observe a significant difference in overall survival (Supplemental Figure 4H), by 6- months after transplantation, the mice became moribund and were sacrificed for analysis. We did not see changes in the multipotent stem cell compartment of the BM (Figure 6K); however, the *Fbxo11*^+/–^ mice showed a reduction in immunophenotypically defined granulocyte-monocyte progenitor (GMP) populations, concomitant with significantly worse neutropenia in the periphery (Figure 6L, Supplemental Figure 4I, and Figure 6M). Lymphocyte, RBC, and platelet counts were not significantly changed by *Fbxo11* in the MDS model (Supplemental Figure 4J).

We tested a second model of MDS using *Nup98-Hoxd13*–transgenic mice (44). We isolated c-Kit^+^ HSPCs and transduced the cells ex vivo with lentiviral vectors encoding shControl (sh*CTRL*) or 1 of 3 independent shRNAs targeting *Fbxo11* (Figure 6N). We expanded a subset of *Fbxo11* targeted cells in methylcellulose and collected them to confirm knockdown of FBXO11. All 3 sh*Fbxo11* samples showed a striking decrease in NPM1, which is consistent with our observation in primary MDS CD34^+^ cells and further supports a model in which ubiquitylation by SCF-FBXO11 maintained NPM1 levels (Figure 6O). In parallel, we transplanted equivalent numbers of shRNA-expressing cells normalized by GFP^+^ reporter percentages and monitored the peripheral blood counts for these groups over time. Mice receiving sh*Fbxo11*-expressing HSPCs presented with significant neutropenia and monocytopenia compared with mice in the control group 2 months after transplantation (Figure 6P), as well as BM hypercellularity (Figure 6, Q and R). There were no differences in lymphocyte, RBC, platelet counts, or overall survival between the sh*CTRL* *Nup98-Hoxd13* recipients and the sh*Fbxo11* recipients (Supplemental Figure 4, K and L). Taken together, our in vitro and in vivo MDS models exhibited the expansion of FBXO11-low MDS HSPCs and demonstrated their contribution to MDS progression through exacerbated neutropenia. By using models of MDS and examining the proteome of primary MDS HSPCs, we observed the loss of NPM1 regulation by the SCF-FBXO11 complex and show that this underlies the poor prognostic features of MDS progression.

### FBXO11 mutations in myeloid malignancies reveal an N-terminal intrisically disordered region.

Given the tumor-suppressive functions of *FBXO11* in hematologic malignancies, we queried whole-exome sequencing data from the Memorial Sloan Kettering Cancer Center (MSK) IMPACT-HEME study for *FBXO11* mutations. While mutations in *FBXO11* have been identified in diffuse, large B cell lymphoma (DLBCL) and Burkitt’s Lymphoma (13, 14), these mutations predominantly occur in the substrate recognition sites on FBXO11: CASH domains from amino acids 418–837. However, *FBXO11* mutations in myeloid malignancies had gone unreported, with the exception of a P45-Q53 deletion in 1 patient in the OHSU Acute Myeloid Leukemia study (https://www.cbioportal.org).

**Figure 7:**
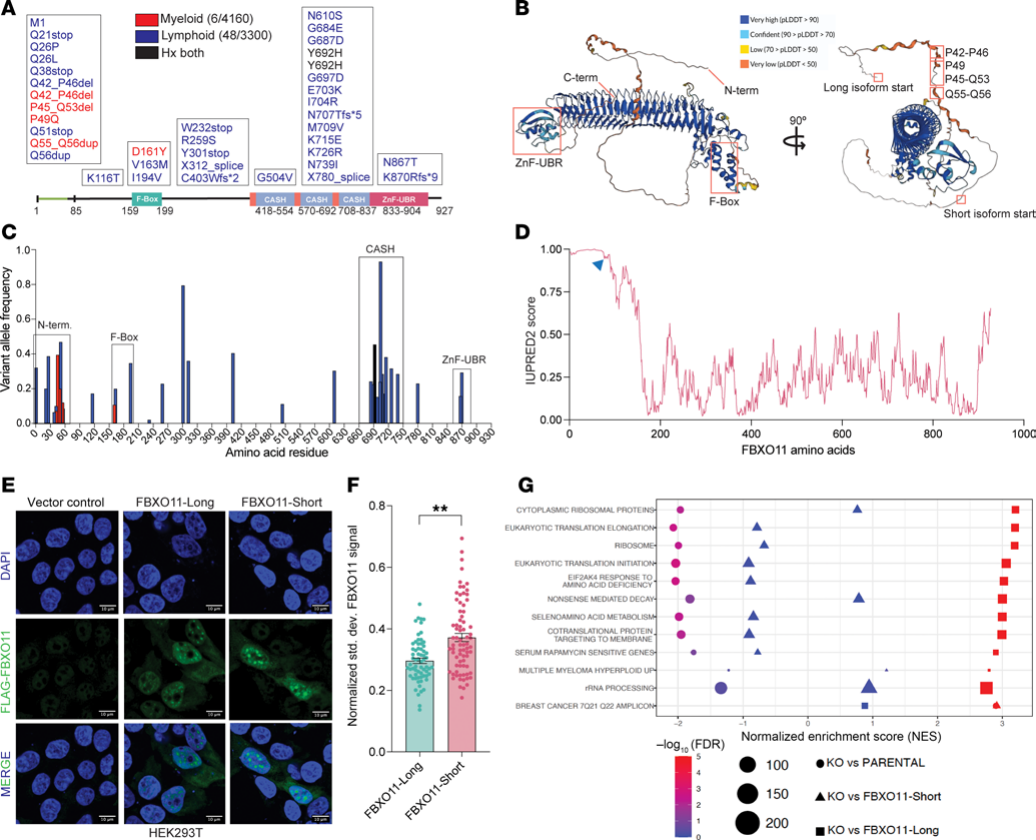
Identification of *FBXO11* mutations in myeloid malignancies reveals a functional N-terminal IDR. (**A**) Map of *FBXO11* mutations identified by whole-exome sequencing of the MSK-IMPACT cohort of patients with hematologic malignancies. (**B**) Alpha-fold predicted structure of FBXO11 with structural features and mutations within myeloid diseases mapped in the disordered N-terminus. (**C**) Variant allele frequencies plotted along the amino acid residues affected by *FBXO11* mutations; FBXO11 functional domains are boxed. N-term., N-terminus; ZnF-UBR, zinc finger-ubiquitin-protein ligase E3 component n-Recognin 1. (**D**) Prediction of intrinsically unstructured proteins 2 (IUPRED2) score of amino acid residues in FBXO11-long. The blue arrowhead indicates the initial methionine residue in FBXO11-short. (**E**) Representative confocal images of FBXO11-long and FBXO11-short expressed in HEK293T cells. Scale bars: 10 μm. (**F**) Quantification of signal distribution of FBXO11-long^+^ and FBXO11-short^+^ cells using the SD of FLAG-FBXO11 signal across each nucleus. **P < 0.01, by 2-tailed, unpaired Mann-Whitney *U* test. (**G**) GSEA analysis of RNA-Seq data (GSE156708) from MDS-L cells that were FBXO11-KO compared with MDS-L parental cells, cells with reexpression of FBXO11-long cDNA, or cells with reexpression of FBXO11-short cDNA. The size of circle indicates the number of genes in the set.

As expected, our analysis revealed *FBXO11* mutations in patients with lymphoid disorders that were within the CASH domains, including the well-characterized Y692H mutation that disrupts BCL6 binding (Figure 7A, blue). Strikingly, we also identified 12 mutations that clustered in the N-terminus of FBXO11 — between residues 1 and 56 — including patients with myeloid malignancies (Figure 7A, red). Using the AlphaFold Protein Structure Database (45), we mapped the myeloid mutations onto the FBXO11 structure (Figure 7B). These mutations mapped to a low-confidence predicted substructure, upstream of the alternative start site at M85 for FBXO11-short (12), which we showed did not efficiently bind NPM1. We plotted the variant allele frequencies of all identified mutations along the linear domain structure of FBXO11 and observed variant allele fractions of the N-terminal mutations equivalent to those in the CASH domain (Figure 7C and Supplemental Figure 5A). We detected N-terminal mutations in patients who had myelofibrosis, myelofibrosis (MF) progressed from myeloproliferative neoplasm (MPN), chronic myelomonocytic leukemia (CMML), or MDS (Table 1) and had no history of a lymphoid malignancy. To test if the mutant alleles coded for protein, we expressed FBXO11 mutants in HEK239T cells and confirmed their predicted molecular weights (Supplemental Figure 5B).

The N-terminal region unique to the FBXO11-long isoform contains small polyglutamine (polyQ) tracts, which are known to exist in intrinsically disordered regions (IDRs). IDRs are defined as regions failing to form stable structures while exhibiting biological properties (46) and affect intramolecular aggregation as well as intermolecular interactions (47). We utilized IUPred2 (48–50) to evaluate the probability of disorder for the amino acid sequences in FBXO11-long (Figure 7D). The first 85 amino acids of FBXO11 upstream of where its short isoform would start were highly probabilistically disordered, with IUPred2 scores approaching 1. To determine whether this novel IDR in FBXO11 could mediate aggregation, we evaluated the staining pattern of Flag-tagged FBXO11-long or FBXO11-short in HEK293T cells by confocal microscopy. In cells that expressed FBXO11-long, we saw a combination of diffuse nucleus-positive signals among less-defined foci (Figure 7E). In contrast, when we expressed FBXO11-short, we detected large, distinct FBXO11 foci in the nucleus of the cells. To quantify this pattern, we analyzed the SD of AF488^+^ signal intensity on a per-pixel basis within each nucleus. FBXO11-short^+^ cells had a significantly greater deviation of FBXO11 intensity relative to the rest of the pixels in a cell (Figure 7F). To understand how the FBXO11 IDR might affect MDS cell states, we reexamined RNA-Seq data from MDS-L cells in our prior studies, in which we knocked out FBXO11 and reexpressed FBXO11-long or FBXO11-short. We compared the gene expression programs of these MDS cells by GSEA. We observed profound changes to pathways affecting the ribosome, metabolism, and RNA processing with FBXO11-long overexpression (Figure 7G), which were consistent with the pathways we saw altered in the primary MDS HSPC proteome. Taken together, we have uncovered a functional N-terminal IDR in FBXO11 that is mutated in myeloid malignancies. We show that this IDR altered intramolecular FBXO11 nuclear organization, facilitated intermolecular NPM1 binding and ubiquitylation, and markedly affected MDS cell gene expression programs.

## Discussion

In exploring the MDS proteome through FBXO11, we uncovered rewiring of protein interaction hubs controlling splicing and ribosome biogenesis. NPM1 is at the center of this hub and is a target of FBXO11-mediated ubiquitylation. Our data suggest that inefficient ubiquitylation of NPM1 caused by suppression of the substrate receptor *FBXO11* contributes to MDS pathogenesis.

Ubiquitylation of NPM1 did not result in its degradation; rather, FBXO11-mediated ubiquitylation of NPM1-K248 permitted the redistribution of NPM1 into the nucleoplasm of normal HSPCs. By qualitatively examining single CD34^+^ cells with confirmed knockdown of *FBXO11* compared with control cells, we observed the redistribution of NPM1 signal into slightly more circular and more fragmented bodies. In primary MDS HSPCs, we saw that both FBXO11 and NPM1 were depleted compared with healthy controls, while in the *Nup98-Hoxd13* murine model, knockdown of *Fbxo11* led to a profound decrease in NPM1. This was consistent with our in vitro data showing that ubiquitylation of NPM1 by the SCF-FBXO11 complex was ultimately protective. We believe our findings are notable because posttranslational modifications of NPM1 controlled its folding, assembly into pentamers, localization, and binding to other proteins (51–53). For example, under normal conditions, p-Thr199 localized NPM1 to nuclear speckles, where it bound RNA structural elements and led to the suppression of pre-mRNA splicing (54, 55). Along with NPM1 and HNRNPU, FBXO11 interactions were markedly enriched for core spliceosome components, RBPs, and ribosomal subunits. Deletion of *FBXO11* led to aberrant exon skipping by splicing reporter assays, and this effect was rescued by NPM1 overexpression. Furthermore, in MDS CD34^+^ cells, low expression levels of *FBXO11* were associated with an alternative splicing program.

Through our quantitative proteomics analysis of HSPCs from patients with MDS compared with those of healthy controls, we discovered that the FBXO11-NPM1 interactome was profoundly perturbed in MDS. Despite our understanding that alternative splicing is a key pathway relevant to MDS, clinical trials for splicing modulators in patients with MDS did not show an improvement in complete or partial response rates (56, 57). Here, we provide evidence that ubiquitylation events mediated by FBXO11 (58, 59) could restructure RNA-binding protein networks in MDS HSPCs, potentially underlying the limited therapeutic window for targeting any one spliceosome component. RNA-binding proteins also link to ribosome biogenesis in MDS (60, 61), and NPM1 is central to this process. Ribosomal subunits in the FBXO11-NPM1 nexus were downregulated in the MDS HSPC proteome, independent of del (5q) or the ribosomal assembly factor mutations observed in other marrow failure disorders (62–64).

Using in vivo murine MDS models driven by *RUNX1*- or *Nup98-Hoxd13*, we found that animals with knockdown of *Fbox11* had exacerbated neutropenia compared with animals with intact *Fbxo11*. This observation is clinically relevant, as neutropenia, combined with anemia, is a prognostic indicator of MDS-related morbidity and transformation to AML (65). In the *RUNX1*-driven transplant experiments, *Fbxo11* deficiency led to expansion of *RUNX1*-mutant clones in the periphery and BM, which would be more consistent with AML transformation than overt marrow failure. Moreover, in the *Nup98-Hoxd13* model, the neutropenia and monocytopenia in *Fbxo11*-knockdown animals were even worse and were accompanied by striking BM hypercellularity and a reduction in NPM1 protein levels. While not formally part of the Revised International Prognostic Scoring System scoring for MDS, BM hypercellularity predicted worse overall survival in some studies, especially those involving patients with de novo MDS (66, 67). Our experimental models demonstrate that FBXO11 deficiency and the resultant shutdown of an NPM1 network portend MDS disease progression.

Mutations in the FBXO11 substrate–binding CASH domains were previously identified in diffuse large B cell lymphoma, and multiple scattered mutations were reported in a rare FBXO11-related neurodevelopmental disorder (68). Here, we screened patients with hematologic malignancies from the MSK-IMPACT-HEME study and uncovered what we believe to be novel *FBXO11* mutations in a previously undiscovered IDR in its N-terminus. Of note, in myeloid diseases, we only observed the rare N-terminal mutations, not those in the CASH domains. This suggests that the N-terminus might have lineage-specific functions in myeloid progenitor cells. Our data show that one function of the FBXO11 IDR is to facilitate the binding and ubiquitylation of NPM1.

Ubiquitin substrate receptor proteins such as FBXO11 have served as surface interfaces for molecular glues, proteolysis targeting chimeras, and induced proximity platforms. By improving our understanding of the biology of these proteins and substrate specificity in hematologic malignancies, we will be able to apply small molecules to direct stem and progenitor cell states as a therapeutic approach for treating aggressive hematologic diseases.

## Methods

### Sex as a biological variable.

Both male and female murine recipients were used in the transplantation experiments. MDS samples included specimens from both male and female individuals. Sex was not analyzed as a biological variable in this study.

### Cell lines and cell culture.

The MDS92 cell line was provided by Daniel Starczynowski (Cincinnati Children’s Hospital Medical Center, Cincinnati, Ohio, USA) and cultured in RPMI with 10% FBS, 1% penicillin-streptomycin, and 10 ng/mL IL-3. The F-36P cell line (American Type Culture Collection [ATCC]) was maintained in RPMI with 20% FBS, 1% penicillin-streptomycin, and 10 ng/mL GM-CSF. Cell line identities were confirmed by short tandem repeat profiling, and all cultures were verified as mycoplasma-negative by PCR. F-36P–Cas9 cells were generated by transduction with Cas9-blasticidin lentivirus followed by blasticidin selection. HEK293T (ATCC) and Plat-E (ATCC) cells were cultured in DMEM supplemented with 10% FBS and 1% penicillin-streptomycin; Plat-E media additionally contained 1 μg/mL puromycin and 10 μg/mL blasticidin.

### CRISPR/Cas9 deletions by electroporation.

FBXO11-deficient cell pools for CRISPR/Cas9 screening were generated by electroporation of sgFBXO11 or sgCTRL constructs into SpCas9^+^ cells using the Lonza 4D Nucleofector X unit and appropriate buffers (P3 for CD34^+^, SF for F-36P, SE for MDS92). All electroporations were performed in 20 μL cuvette strips per the manufacturer’s protocol. gRNAs with modified scaffolds (Synthego) were mixed with 3×NLS-SpCas9 (St. Jude Protein Production Facility) at a 3:1 molar ratio; for multiplexing, the 3× ratio was divided among all gRNAs. For example, 1 gRNA = 1.5 μL gRNA (100 μM stock) plus 1 μL Cas9 (50 μM); 2 gRNAs = 0.75 μL each gRNA plus 1 μL Cas9. The electroporation programs were DN-100 for MDS92 and EH-100 for F-36P–Cas9 cells.

### Animal models and comparative pathology.

BM cellularity in transplanted mice was evaluated by blinded analysis of H&E-stained femur sections. *Fbxo11*^tm1a^ ^(EUCOMM)Wtsi^ mice were obtained from Eucomm IMPC and established at St. Jude by Peng Xu and Mitchell Weiss. Mice were first crossed with *FlpO*^+^ mice to delete the lacz reporter cassette and then with *Mx1-Cre^+^* mice to generate the conditional allele model (Figure 5). Genotyping of the Tm1A *Fbxo11* allele was performed using tail genomic DNA. *Mx1-Cre^+^* (strain no. 003556) and *Nup98-Hoxd13* (strain no. 010505) mice were from The Jackson Laboratory. All donors were C57BL/6J (CD45.2^+^, strain no. 000664); recipients were B6.SJL-Ptprca Pepcb/BoyJ (CD45.1^+^, strain no. 002014).

### BM transplantation.

Donor cells were isolated from the tibia, femur, iliac crest, and spine of *Fbxo11^fl/+^* *Mx1-Cre^+^* mice and enriched for HSPCs using the CD117 magnetic bead isolation kit (catalog 130-091-224, Miltenyi Biotec). Cells were stimulated overnight and then transduced with the indicated retro- or lentiviruses. Recipient mice (CD45.1^+^) were conditioned for transplantation using 9.5 Gy irradiation prior to tail vein injection of donor cells in 100 μL PBS.

### Virus production and transductions.

*Runx1* retroviral constructs were provided by Dong-Er Zhang (UCSD, San Diego, California, USA). Mouse shRNA constructs targeting *Fbxo11* were purchased from Origene (pLenti-GFP vector). Human lentiviral shRNA constructs targeting FBXO11 in the pLVRU6MP-mCherry vectors were purchased from Genecopoeia. Lentiviruses were produced in VSVG packaging by the St. Jude Viral Vector Core. Retrovirus was produced by transfecting Plat-E cells with 10 μg transfer plasmid with pMD2.G in 6 mL per 10 cm dish, seeded at 5 × 10^6^ cells/10 cm plate in DMEM with 10% FBS, 1 μg/mL puromycin, and 10 μg/mL blasticidin. Prior to transfection, culture media were removed and replaced with 5 mL prewarmed Opti-MEM. The transfection reaction was 1 mL Opti-MEM with no serum, 10 μg MigR1-RUNX1-41-214 plasmid, and 20 μL Turbofect, incubated for 15–20 minutes at room temperature, and then carefully transferred to plates dropwise followed by overnight incubation. Transfection media were replaced with 5 mL Opti-MEM with serum and incubated for 24 hours. Supernatant was collected at 48 hours. Media containing retroviruses were collected and filtered through a 0.4 μm filter and used fresh on isolated c-Kit^+^ cells. Viral supernatant (3 mL) was added to c-Kit^+^ cells and then spinoculated at 300 *g* for 1.5 hours at 32°C. Cells were incubated for 1–2 hours at 37°C, and then media were replaced and cells were spinoculated a second time with an additional 3 mL viral supernatant before being incubated overnight at 37°C.

### Flow cytometry and immunophenotyping.

Peripheral blood and BM cells from tibias, femurs, and pelvic bones were collected, and the samples were RBC lysed, washed, and resuspended in FACS buffer (PBS/2% FBS) and analyzed on a BD Symphony A3. BM hematopoietic progenitors were stained with immunophenotyping panels as previously described (69). All flow cytometric data were analyzed using FlowJo, version 10.

### Generation of the CRISPR-KO library and KO screen analysis.

In brief, the CRISPR-KO library was first designed using CRISPick (70, 71). Oligonucleotides were synthesized by TWIST Bioscience. Library amplification and Gibson Assembly into the LRG vector (72) (Addgene, no. 65656) backbone was performed according to the method of Joung et al. (73). Validation of sgRNA representation was performed using calc_auc_v1.1.py (https://github.com/mhegde/) and count_spacers.py (73). MaGeCK-VISPR/0.5.7 (74, 75) was used to identify guides and genes that were significantly depleted or enriched in FBXO11-KO versus control cells. Detailed methods can be found alongside the data in Supplemental Table 2.

### Cell lysis and nuclear fractionation.

F-36P and HEK293T cells were washed with PBS and harvested by trypsinization. Cells were pelleted and resuspended in cold buffer A (20 mM HEPES pH 7.4, 10 mM KCl, 0.2 mM EDTA) with protease inhibitors. Cytosolic fractions were obtained by adding 10% NP-40, followed by brief vortexing and centrifugation (14,000 rpm, 30 seconds, 4°C). Nuclear pellets were washed with buffer A, lysed in cell lysis/IP buffer (20 mM phospho-buffer pH 7.5, 150 mM NaCl, 10% glycerol, 0.2% Triton X-100), and incubated at 4°C for 20 minutes with rotation. Insoluble chromatin fractions were digested with DNase (Thermo Fisher Scientific, 88700) and clarified by centrifugation. Nuclear soluble and insoluble fractions were combined for IP.

### Purification and proteome profiling of FBXO11 complexes.

The immunoprecipitated FBXO11-interacting proteins described above were identified by proteome profiling with spectral counting at the St. Jude Proteomics Core Facility as previously described (76, 77). Detailed methods can be found alongside these data in Supplemental Table 1.

### RNA expression analyses and rMATs of samples from patients with MDS.

FBXO11 expression in MDS CD34^+^ cells with or without splicing factor mutations was analyzed using the Gene Expression Omnibus (GEO) dataset GSE58831 (37). Samples in the upper (75%) and lower (25%) quartiles of FBXO11 expression were designated FBXO11-high and FBXO11-low, respectively. Replicate multivariate analysis of transcript splicing (rMATS) was used to identify significant alternative splicing events (FDR < 0.05, *P* < 0.05, |inclusion level difference| >0.15) compared with healthy controls.

### Splicing reporter assay.

F-36P–Cas9 cells were transfected with sgCTRL or sgFBXO11, followed by electroporation with 1 μg RG6 splicing reporter plasmid (Addgene, no. 80167). Cells were incubated for 24–48 hours, and reporter activity was quantified by flow cytometry. HEK293T cells were transiently cotransfected with RG6 reporter and FLAG-tagged FBXO11 isoforms using TransIT-LT1, and reporter expression was analyzed 48 hours after transfection.

### Processing of MDS CD34^+^ patient samples and data-independent acquisition proteomics analysis.

Total BM cells from patients with MDS were obtained under IRB-approved protocols at Northwestern University and Albert Einstein College of Medicine. In brief, data-independent acquisition (DIA) data were analyzed using DIA-NN, version 1.8, as previously described (78, 79) and quantified using the MaxLFQ algorithm implemented in the iq r package (80). Detailed methods can be found alongside these data in Supplemental Table 3.

### NPM1 co-IP and ubiquitylation assays.

HEK293T cells were cotransfected with FBXO11 isoforms and ubiquitin-eGFP (Addgene, no. 187910) using TransIT-LT1. Nuclear lysates were immunoprecipitated with anti-NPM1 (Thermo Fisher Scientific, 32-5200) conjugated to Protein A/G magnetic beads (Thermo Fisher Scientific, 88803). Bound complexes were eluted in 2× SDS and analyzed by Western blotting using anti-FBXO11 (Novus Biologicals, NB100-59826), anti-NPM1 (Cell Signaling Technology [CST], 92825S), and anti-GFP (Thermo Fisher Scientific, A-11122).

### CD34^+^ cell confocal microscopy.

CD34^+^ cells were prepared on slides by cytospin, fixed in 4% paraformaldehyde, permeabilized with 0.1% Triton X-100, and blocked with 5% BSA. Cells were incubated with primary antibodies (NPM1 4 μg/mL, FBXO11 10 μg/mL) overnight at 4°C, followed by fluorophore-conjugated secondary antibodies (Alexa Fluor 488/647, 1:1,000; Thermo Fisher Scientific). Nuclei were stained with DAPI (0.1 μg/mL). Images were captured on a Zeiss 780 confocal microscope with a ×63 oil objective. Settings were reused for images. Colocalization analysis was performed using macros in FIJI applied to raw images with NPM1 (green) and FBXO11 (far red) channels. Pearson correlation values for FBXO11 and NPM1 were determined in each cell. NPM1-bright and NPM1-dim nuclear compartments were determined by nuclear regions of interest (ROIs) around the DAPI channel, applying masking according to the automatic/relative thresholding of NPM1 within nuclei. Images were pseudocolored by look-up tables. The same masking was applied to identify NPM1-bright objects and shape descriptors. The aspect ratio was calculated using the formula AR = minFeret/maxFeret.

### ChIP.

MDS92 and F-36P cells (10^7^ per replicate, *n* = 3) were crosslinked with 1% formaldehyde, lysed in SDS buffer, and sonicated to 200–500 bp fragments. Chromatin was diluted and incubated with anti-MYC (CST, 9402S) or IgG (CST, 2729S) overnight, followed by Protein A/G bead capture. Sequential washes were performed with low-salt, high-salt, and LiCl buffers, followed by elution and crosslink reversal. DNA was purified via phenol/chloroform extraction and ethanol precipitation, resuspended in Tris-EDTA, and analyzed by quantitative PCR (qPCR).

### CD34^+^ cell fitness assay.

One million CD34^+^ cells were transiently transfected using CRISPR cell fitness assay protocols as previously described (42, 81). The Lonza 4D-Nucleofector X-unit was used for electroporation with solution P3 and the program EH-100 in a small (100 μL) cuvette. Next-generation sequencing (NGS) was performed using CRIS.py (82) to determine the indel frequencies. Out-of-frame indels are shown in plots.

### Whole-exome sequencing of FBXO11 mutations.

Patient specimens were sequenced under retrospective protocol 15-017 (IMPACT-HEME study, Memorial Sloan Kettering Cancer Center). All samples were deidentified.

### Statistics.

A 2-sample *t* test or Mann-Whitney *U* test was used to compare 2 groups, based on the normality of the data. All tests were 2 tailed, and data are presented as the mean ± SD unless otherwise stated in the figure legends. A linear regression model was applied to examine the relationship between genetic groups and continuous outcomes, with the donor included as the cluster variable, as appropriate. An additive genetic model was used. A linear mixed-effects model was used to evaluate the effect of time on continuous outcomes, adjusting for linear, quadratic, and cubic terms of time as needed, with mice or wells as the random effect. The Shapiro-Wilk test was conducted to assess normality for the raw data or residuals. All *P* values were 2 sided. To correct for multiple comparisons, as appropriate, the FDR adjusted *P* values (*Q* values) were calculated using the Benjamini and Hochberg method and controlled at 0.05 (83). R–4.4.0 was used to conduct statistical analyses.

### Study approval.

Animal experiments were approved by the St. Jude Children’s Research Hospital IACUC and conducted in AAALAC-accredited facilities. Human specimens were obtained with informed consent under IRB approval from Northwestern University, Albert Einstein College of Medicine, and Memorial Sloan Kettering Cancer Center.

### Data availability.

Proteomics data have been deposited in the PRoteomics IDEntification (PRIDE) database under accession numbers PXD059844 and PXD059857. Supporting data are provided in the Supporting Data Values file. Other primary data are available from the corresponding author.

## Author contributions

MN designed studies, conducted experiments, acquired data, analyzed data, and wrote the manuscript. LB conducted experiments, acquired data, and analyzed data. AC conducted experiments and acquired data. LEP analyzed data. TH designed experiments, conducted experiments, and acquired data. LK designed studies, conducted experiments, and acquired and analyzed data. VRP acquired and analyzed data. JZ conducted experiments and acquired and analyzed data. ZFY, CW, ABT, SG, LJJ, GK, and YF analyzed data. JAS and SN conducted experiments and acquired data. AS and EM acquired data. SA, MS, PX, PJ, MJW, AV, and SMPM provided reagents. AAH designed research studies and provided reagents. RKR acquired data. JDC designed studies, analyzed data, provided reagents, and wrote the manuscript.

## Funding support

This work is the result of NIH funding, in whole or in part, and is subject to the NIH Public Access Policy. Through acceptance of this federal funding, the NIH has been given a right to make the work publicly available in PubMed Central.

- NIH grant R35 CA253096 (to JDC).
- NIH grant P30 CA021765 (to the St Jude Comprehensive Cancer Center).
- NIH grant K00CA234924 (to MN).
- ALSAC/St. Jude Children’s Research Hospital.
- Edward P. Evans Foundation (to MN).

## Supplementary Material

Supplemental data

Unedited blot and gel images

Supplemental table 1

Supplemental table 2

Supplemental table 3

Supplemental table 4

Supplemental table 5

Supporting data values

## Figures and Tables

**Table 1:**
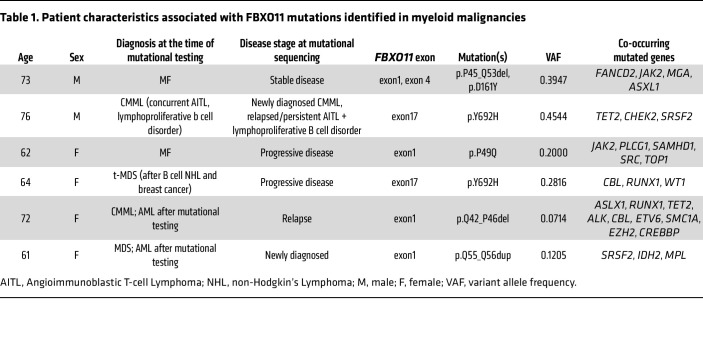
Patient characteristics associated with FBXO11 mutations identified in myeloid malignancies

## Version 1 01/16/2026

Electronic publication
