# Supplementary material for: FBXO11 suppression rewires an NPM1-centered interactome influencing the progression of myelodysplastic syndrome
Source: J Clin Invest. 2026 Jan 16;136(2):e193636. doi: 10.1172/JCI193636 (PMC12807477; doi:10.1172/JCI193636)
Supplement: Supplemental data [file jci-136-193636-s042.pdf]

Supplemental Figure 1.

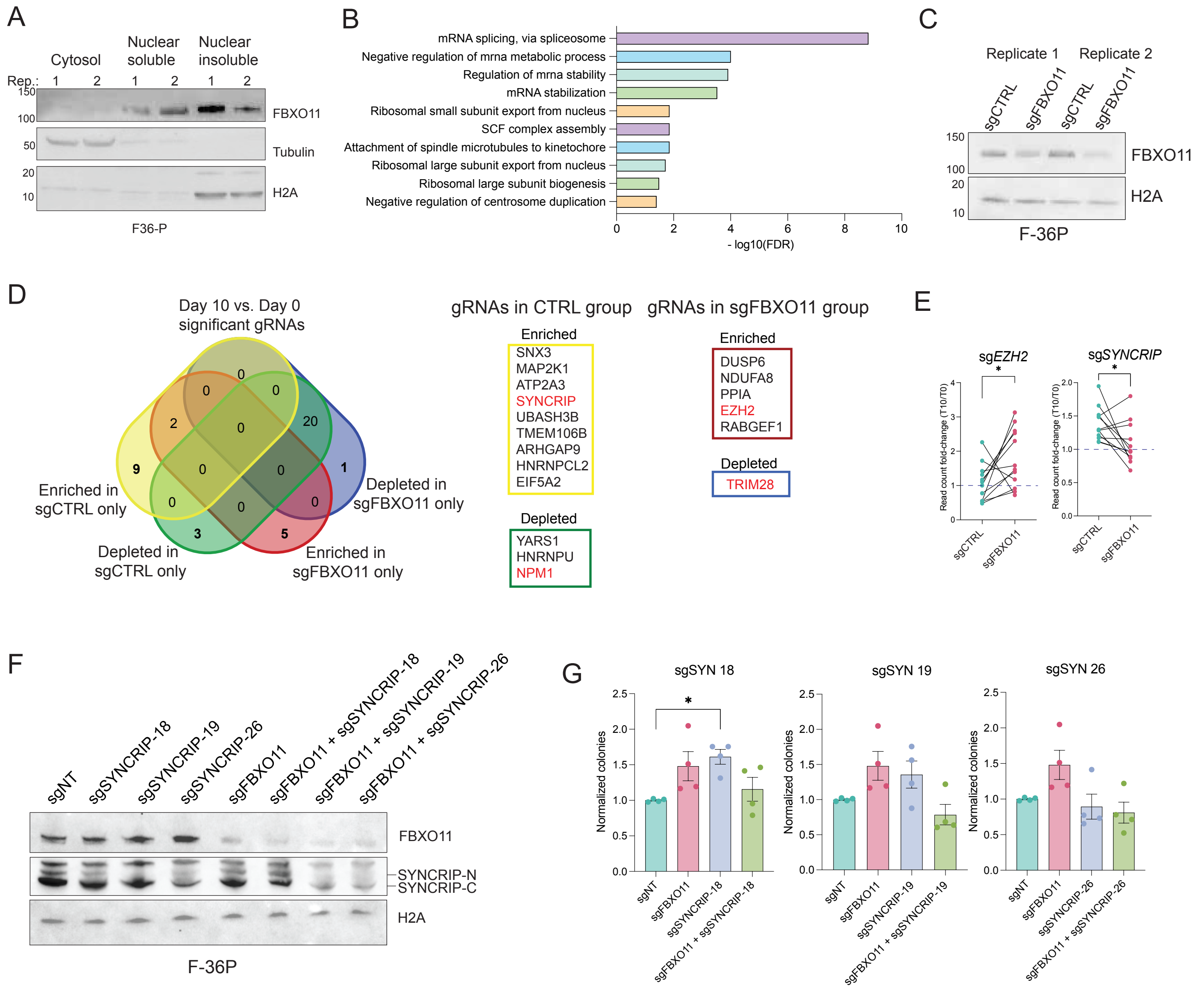

Supplemental Figure 1. (A) Immunoblot of FBXO11, Tubulin, and H2A in cytosolic and nuclear fractions of two biological replicates from F-36P cells. (B) KEGG pathway enrichment performed on FBXO11-interacting proteins, shown are -log10(FDR) values. (C) Immunoblot of FBXO11 and H2A in F-36P-Cas9+ cell pools that were electroporated with sgCTRL or sgFBXO11. Two biological replicates were used for the substrate-focused CRISPR-Cas9 screen. (D) Venn diagram of significant hits in each experimental group in the FBXO11 substrate-focused CRISPR-Cas9 screen in F-36P-Cas9+ cells. Boxes list significantly enriched and depleted guide RNAs for the sgFBXO11 and sgCTRL experimental groups. Guides significantly enriched or depleted in both experimental groups were not dependent on FBXO11 and therefore excluded from the gene lists. (E) Fold-change in individual gRNA read counts for EZH2 and SYNCRIP in the colony-forming assay (Day 10) versus initial representation (Day 0). The fold-change for individual guides was compared between sgCTRL and sgFBXO11 experimental groups, shown on the graphs. n=6 guides per gene, in two independent biological replicates of the CRISPR screen. Two-tailed, paired T test. *P<0.05. (F) Western blot of SYNCRIP and FBXO11 with H2A loading control in F-36P cells electroporated with indicated gRNAs from the CRISPR screen. (G) Normalized colony counts for SYNCRIP guides in independent assays; sgSYNCRIP sensitizes F-36P cells to sgFBXO11, resulting in a partial rescue of colony-forming ability. *P<0.05.

Supplemental Figure 2.

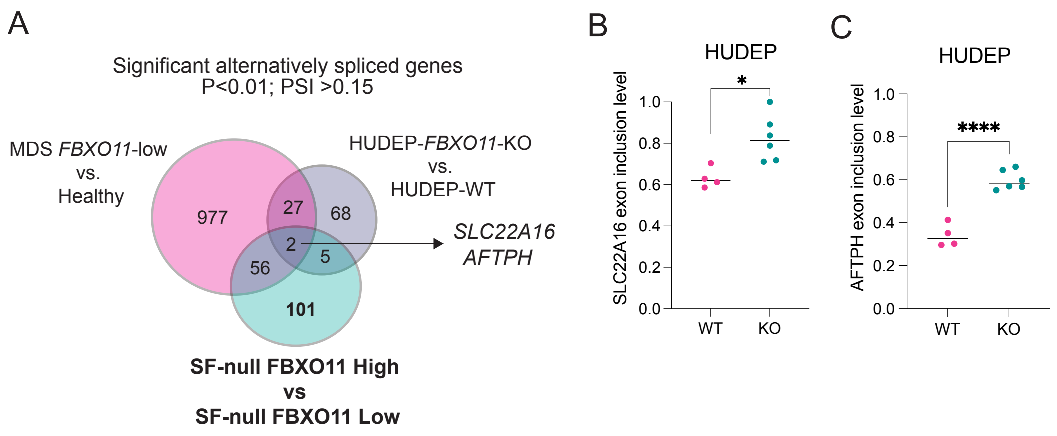

Supplemental Figure 2. (A) Venn diagram of overlapping and distinct alternatively spliced genes from three experimental comparisons: MDS samples with low expression versus healthy, FBXO11 high MDS samples versus FBXO11 low MDS samples that have no splicing factor mutations, and previously sequenced HUDEP-FBXO11-KO versus FBXO11-WT normal erythroid progenitor cells as a control for FBXO11-associated events. SLC22A16 and AFTPH genes were common to all 3 comparisons, representing putative FBXO11-dependent splice events. (B-C) Exon-inclusion levels for SLC22A16 and AFTPH in the experimental groups indicated, FBXO11 WT and FBXO11 KO. For HUDEP graphs, each dot represents a technical replicate from the cells used for RNA-sequencing.

Supplemental Figure 3.

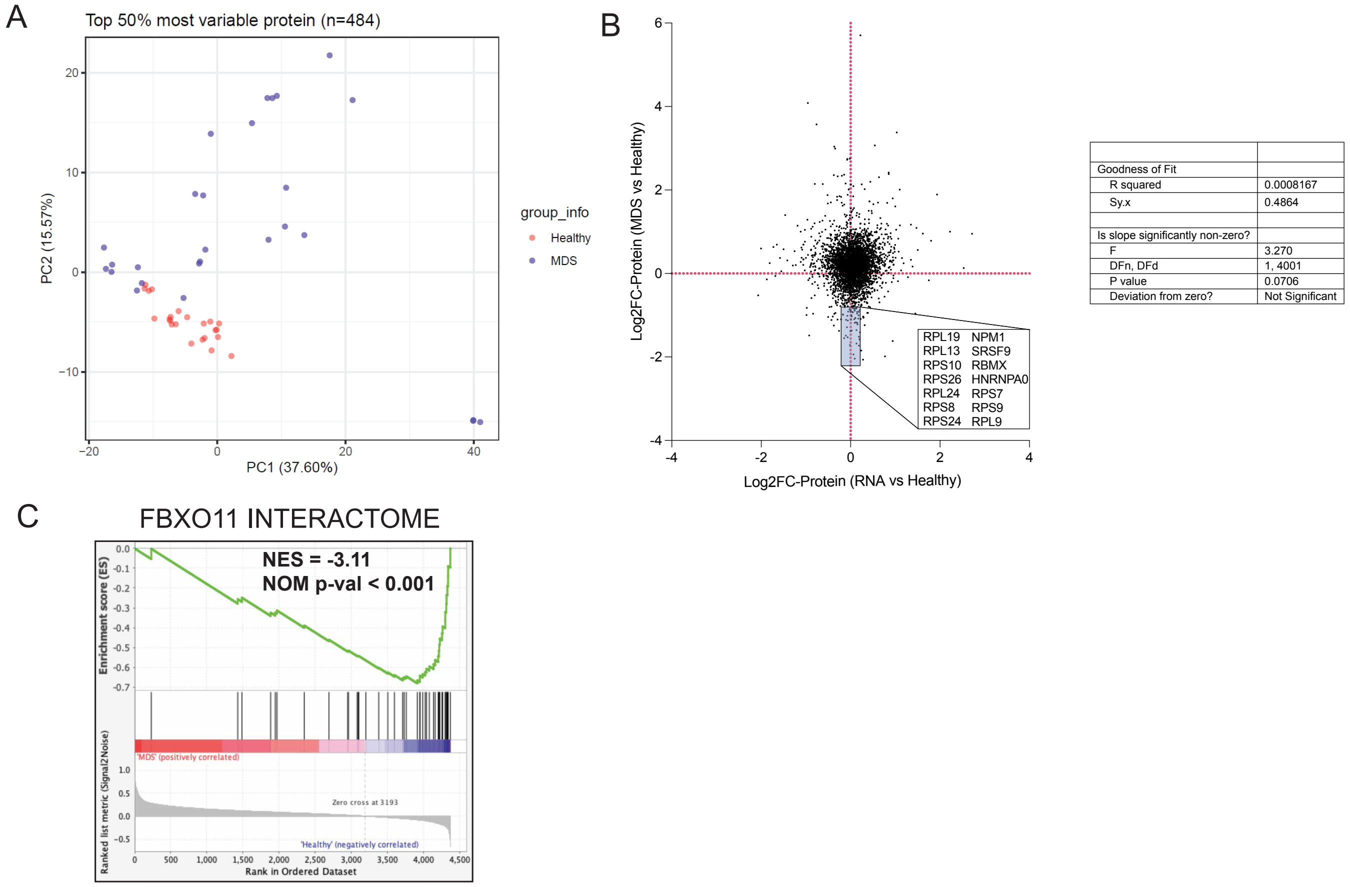

Supplemental Figure 3. (A) Principal component analysis plot of all MDS and healthy control replicate samples analyzed by proteomics. (B) Scatter plot of protein fold-change versus transcript fold-change for proteins and genes detected as differentially expressed in MDS versus healthy CD34+ cells. RNA fold-change was calculated by comparing MDS samples to healthy control samples analyzed from the publicly available bulk-RNA sequencing dataset GSE58831. Datasets were integrated using R merge functions. Resulting scatterplot is shown with Pearson r correlation values below the plot. (C) Gene-set enrichment analysis evaluating the FBXO11 interactome within the MDS proteome. NES = -3.11, P<0.000.

Supplemental Figure 4.

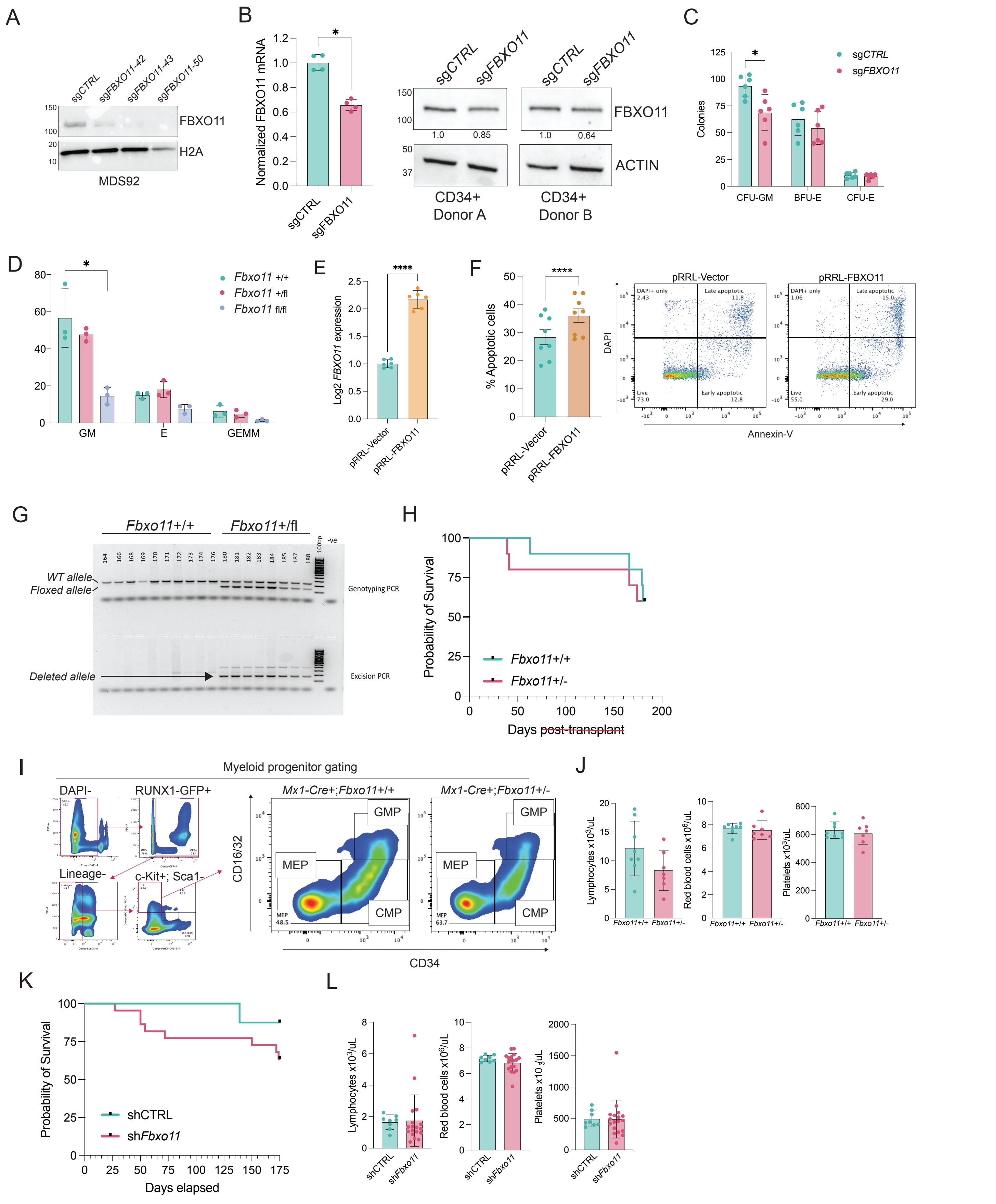

Supplemental Figure 4 (A) Immunoblot of FBXO11 and H2A (loading control) in MDS92 cells electroporated with Cas9 and either control gRNA or gRNAs targeting FBXO11. (B) Left; mRNA expression for FBXO11 in CD34+ cells electroporated with Cas9 and sgCTRL or sgFBXO11, determined by qPCR. Z-test from the coefficient test based on Linear regression model adjusting for donor effect as a cluster variable. n=2 biological CD34+ donors, 2 technical replicates each qPCR assay. Right; western blot of FBXO11 and ACTIN in 2 independent CD34+ donor samples that were electroporated for control or FBXO11-targeting RNPs with Cas9. Values below FBXO11 blots are the densitometry values normalized to the sgCTRL group within each donor. Densitometry was calculated using the plot lanes function in FIJI. (C) Number of colonies formed per 1,000 healthy donor CD34+ cells plated from two independent donors, 3 wells per donor. T-tests and Mann-Whitney tests were applied to compared the two groups depending on the normality of the data, with pooled replicates and corrected for multiple comparisons. *Q<0.05. (D) Number of colonies of murine c-kit+ HSPCs per 1,000 cells in M3434 myeloid methocult. One donor mouse per genotype, 3 wells per mouse. Cells were electroporated with Cre mRNA to delete *Fbxo11* ex vivo. T-tests and Mann-Whitney tests were applied to compared the each group to the WT control, depending on the normality of the data, corrected for multiple comparisons. *Q<0.05, **Q<0.01. (E) FBXO11 expression determined by qPCR in F-36P cells transduced with control pRRL-Vector or pRRL-FBXO11. N = two independent experiments, 3 technical replicates per qPCR assay. Unpaired, two-tailed t-test of pooled replicates. ****P<0.0001. (F) Percent of Annexin-V positive F-36P cells that were transduced with pRRL-Vector or pRRL-FBXO11. n=3 independent experiments, 2-3 wells of cells per group. Unpaired, two-tailed t test with matching was applied. Matched P-value is indicated. ****P<0.0001. Representative flow plots showing DAPI and Annexin-V stained cell populations are shown at the right. (G) Annotated DNA gel image from allele genotyping PCR reactions and excision PCR genotyping reactions at 1-month post-pIpC injections of the mice indicated. (H) Kaplan-Meier survival of the retroviral RUNX1 transplant recipients. No significant difference was observed. End of curve represents termination of experiment at MDS stage, where surviving mice were analyzed. (I) Flow cytometry gating strategy for the analysis of myeloid progenitor cell populations and representative flow plots from Fbxo11+/+ and Fbxo11+/fl groups. (J) Differential complete blood cell counts counts in RUNX1 transplant mice at 16 weeks post-pIpC injections. Unpaired tests were performed. T was used for groups with normal distribution, Mann-Whitney was used otherwise. (K) Kaplan-Meier survival of Nup98-Hoxd13 transplant recipients receiving cells expressing shCTRL or one of three shFbxo11 constructs. P=0.22 determined by Log-rank (Mantel-Cox) test. Survival curve was terminated at the end of the experiment, at which point mice were euthanized due to severe cytopenias. (L) Differential complete blood cell counts in Nup98-Hoxd13 transplant mice at 2 months post-transplant. ShFbxo11 groups were pooled. Unpaired tests were performed. T was used for groups with normal distribution, Mann-Whitney was used otherwise.

Supplemental Figure 5.

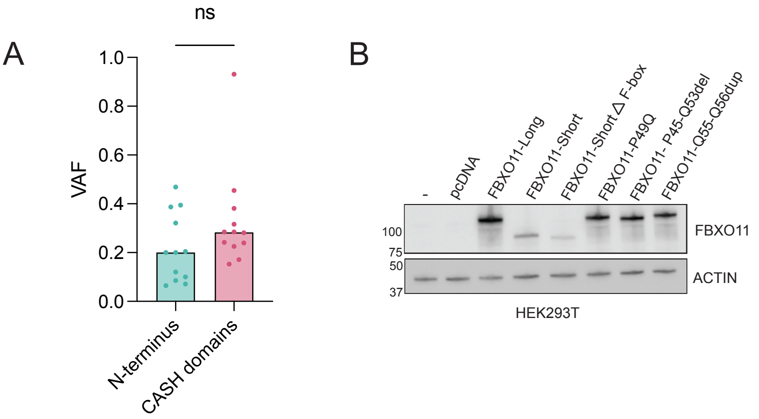

Supplemental Figure 5. (A) Variant allele fractions plotted for mutations in the N-terminus of FBXO11 and mutations in the CASH domain of FBXO11. No significant difference in VAFs observed in these mutated regions. (B) Immunoblot of exogenously expressed FBXO11 isoforms and mutants in HEK293T cells with ACTIN as a loading control.
