# Supplementary material for: FBXO11 suppression rewires an NPM1-centered interactome influencing the progression of myelodysplastic syndrome
Source: J Clin Invest. 2026 Jan 16;136(2):e193636. doi: 10.1172/JCI193636 (PMC12807477; doi:10.1172/JCI193636)
Supplement: Unedited blot and gel images [file jci-136-193636-s043.pdf]

Unedited blot for S1A - FBXO11

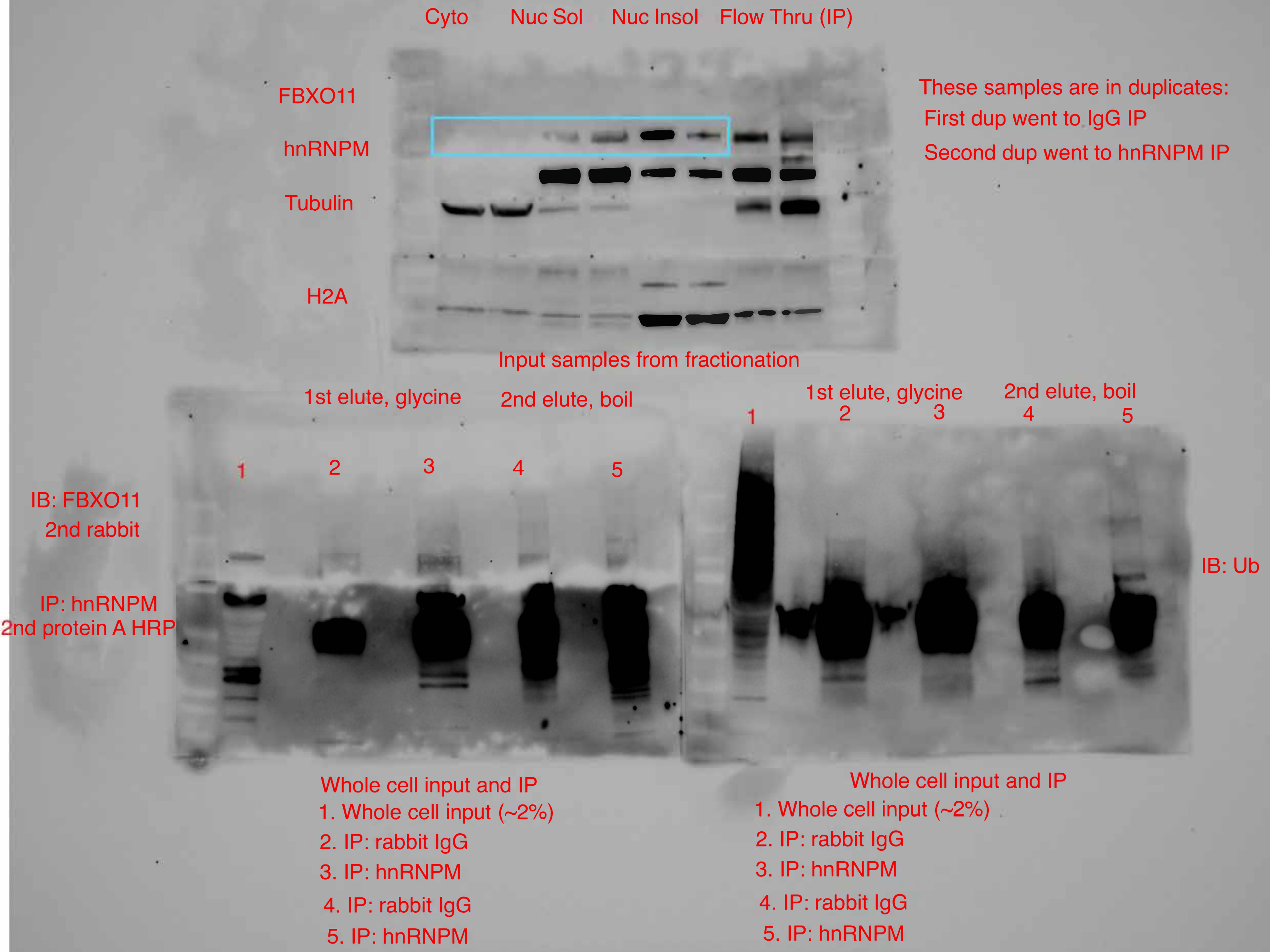

Unedited blot for S1A - Tubulin, H2A

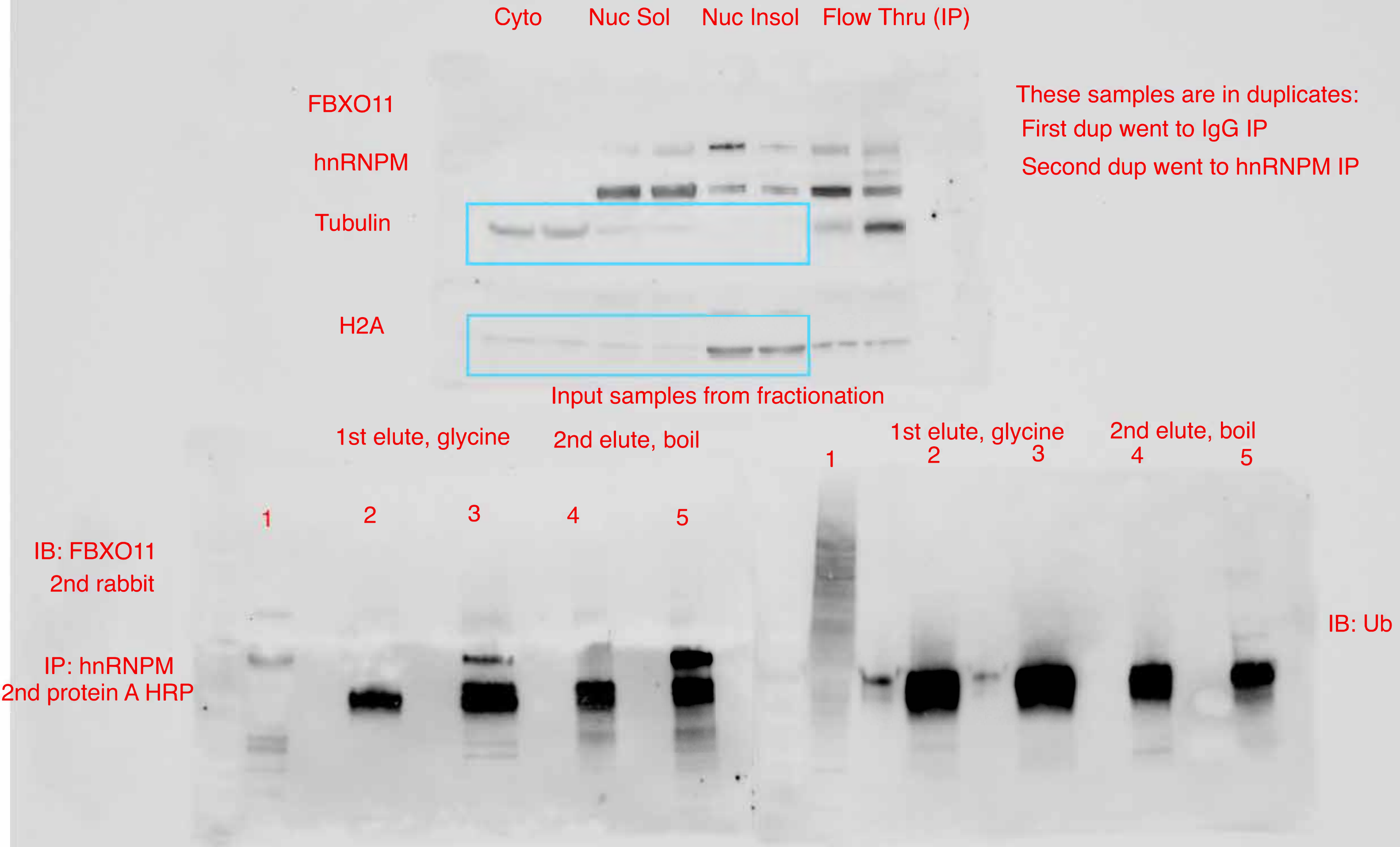

Whole cell input and IP

1. Whole cell input (~2%)
2. IP: rabbit IgG
3. IP: hnRNPM
4. IP: rabbit IgG
5. IP: hnRNPM

Whole cell input and IP

1. Whole cell input (~2%)
2. IP: rabbit IgG
3. IP: hnRNPM
4. IP: rabbit IgG
5. IP: hnRNPM

Unedited blot for Supp Figure [redacted]

1C

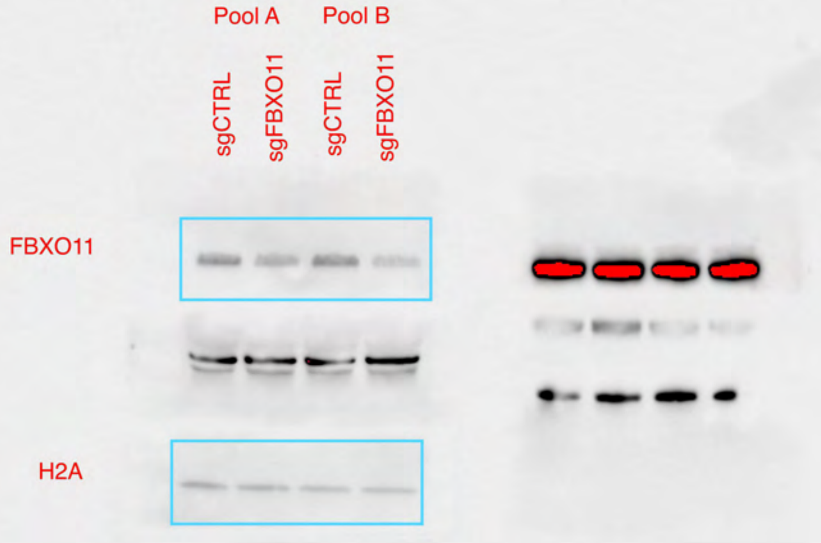

Unedited blot for Supp Figure 1F - FBXO11

1. sgNT
2. sgSYNCRIP-18
3. sgSYNCRIP-19
4. sgSYNCRIP-26
5. sgFBXO11
6. sgFBXO11+sgSYNCRIP-18
7. sgFBXO11+sgSYNCRIP-19
8. sgFBXO11+sgSYNCRIP-26

Text

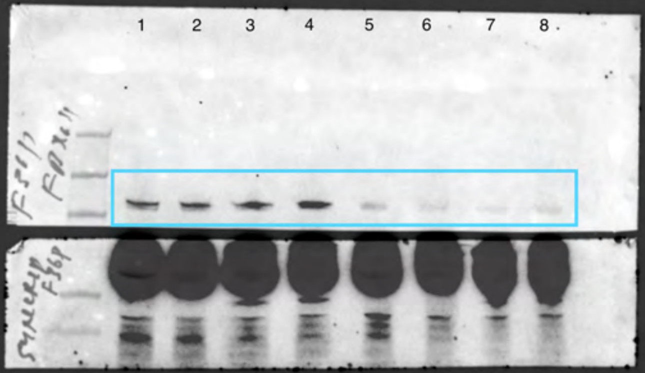

Unedited blot for Supp Figure 1F - H2A

1. sgNT
2. sgSYNCRIP-18
3. sgSYNCRIP-19
4. sgSYNCRIP-26
5. sgFBXO11
6. sgFBXO11+sgSYNCRIP-18
7. sgFBXO11+sgSYNCRIP-19
8. sgFBXO11+sgSYNCRIP-26

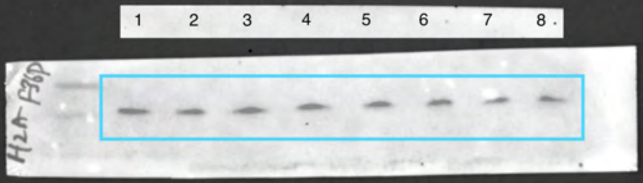

Unedited blot for Supp Figure 1F - SYNCRIP

1. sgNT
2. sgSYNCRIP-18
3. sgSYNCRIP-19
4. sgSYNCRIP-26
5. sgFBXO11
6. sgFBXO11+sgSYNCRIP-18
7. sgFBXO11+sgSYNCRIP-19
8. sgFBXO11+sgSYNCRIP-26

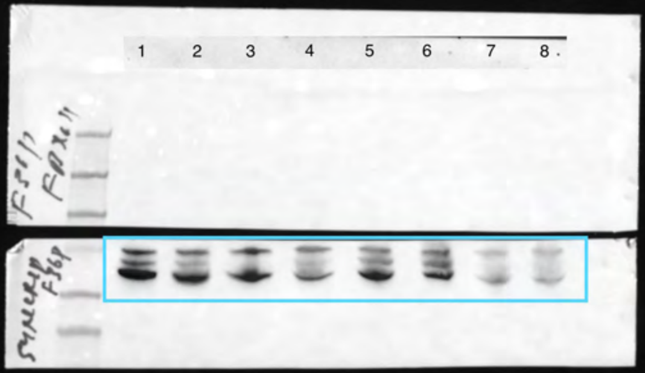

Unedited blot for Figure 2A- FBXO11 input

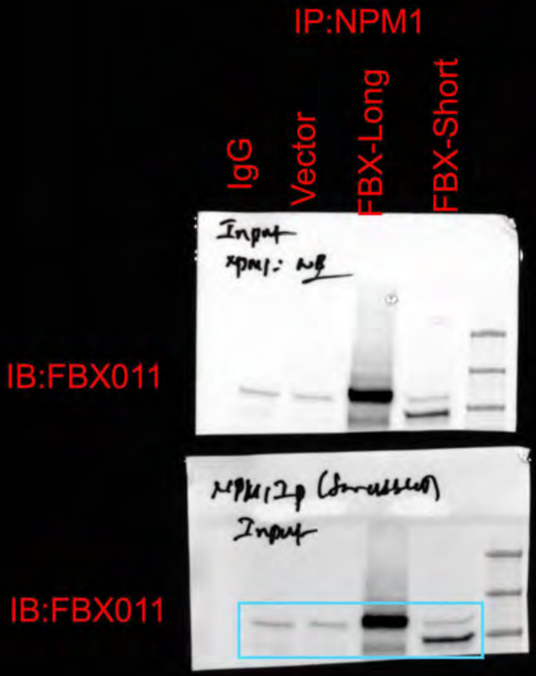

Unedited blot for Figure 2A - H2A input

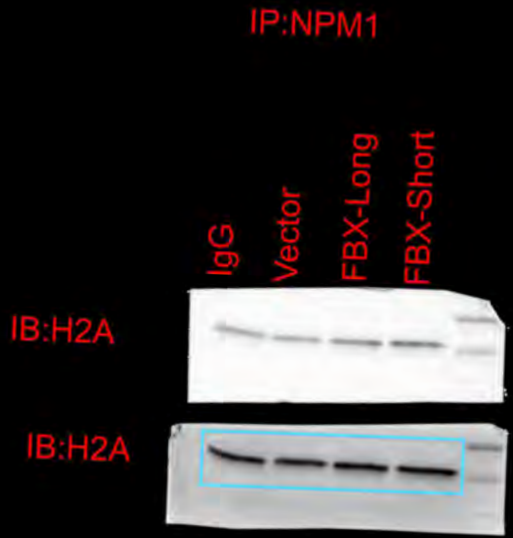

Unedited blot for Figure 2A - NPM1 input

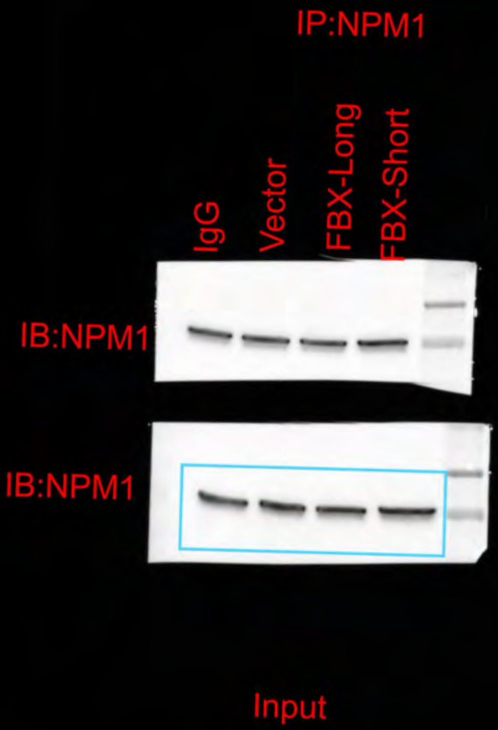

Unedited blot for Figure 2A - FBXO11, IP

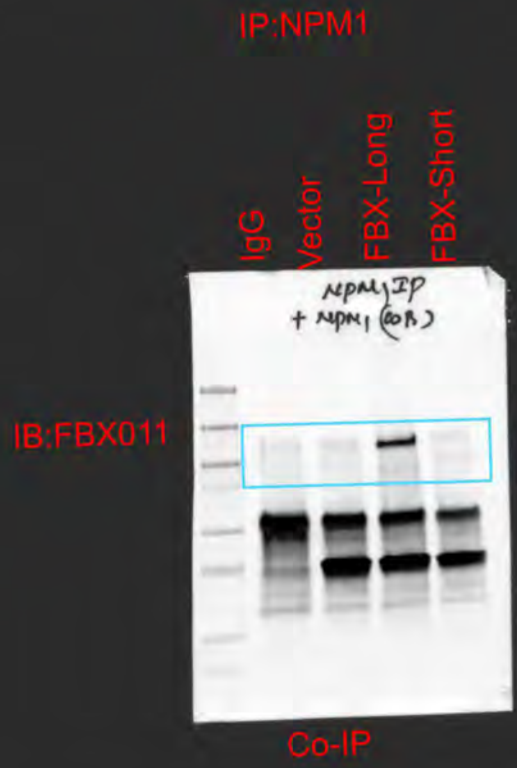

Unedited blot for Figure 2A - NPM1, IP

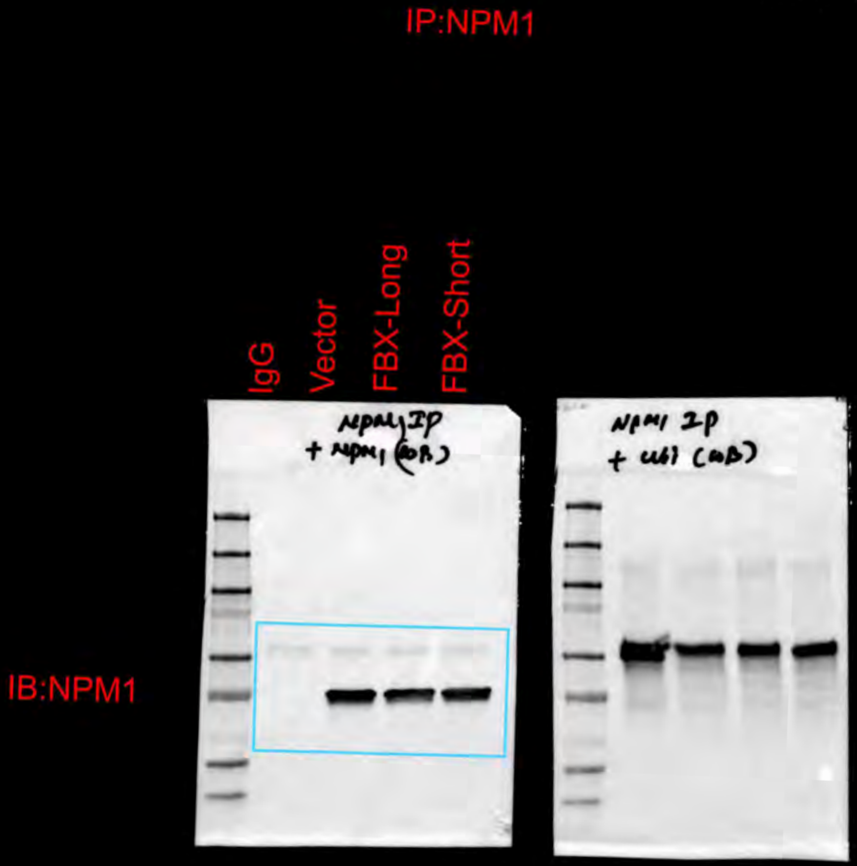

Unedited blot for Figure 2B - FBXO11

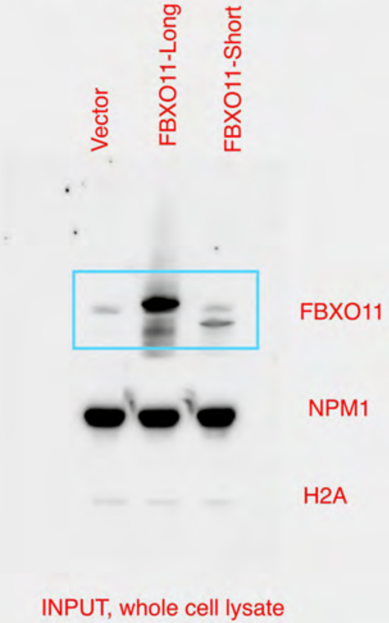

Unedited blot for Figure 2B - NPM1

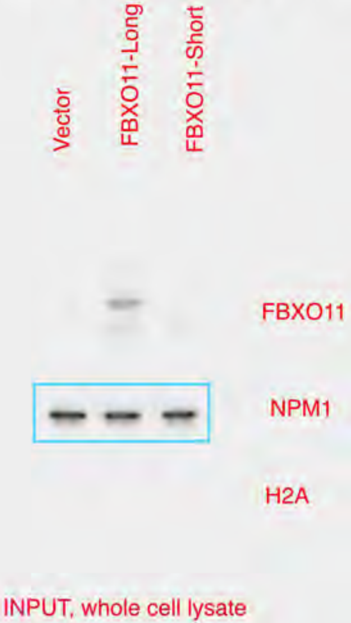

Unedited blot for Figure 2B - H2A

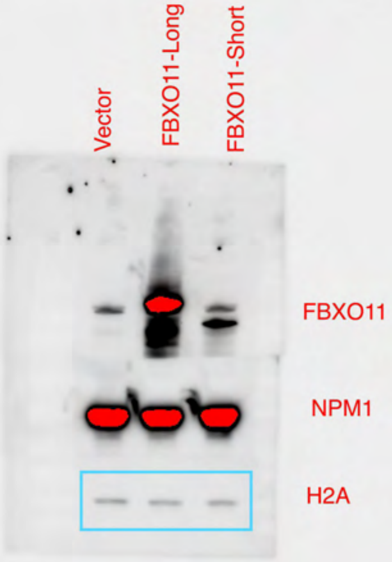

INPUT, whole cell lysate

Unedited blot for Figure 2B - FBXO11, IP

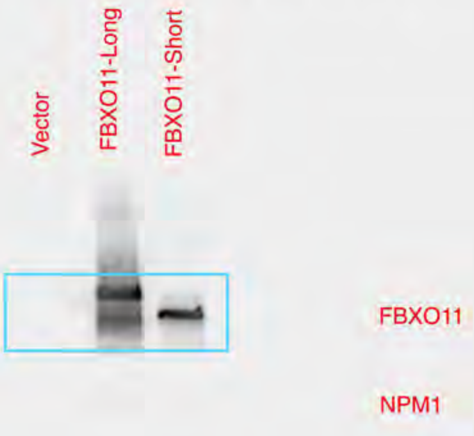

IP: FLAG-FBXO11, blot NPM1

Unedited blot for Figure 2B - NPM1, IP

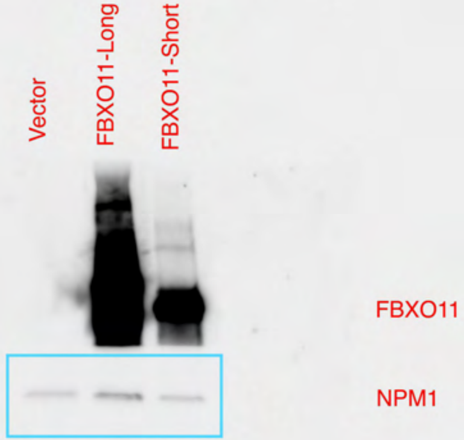

IP: FLAG-FBXO11, blot NPM1

Unedited blot for Figure 2C - FBXO11, input

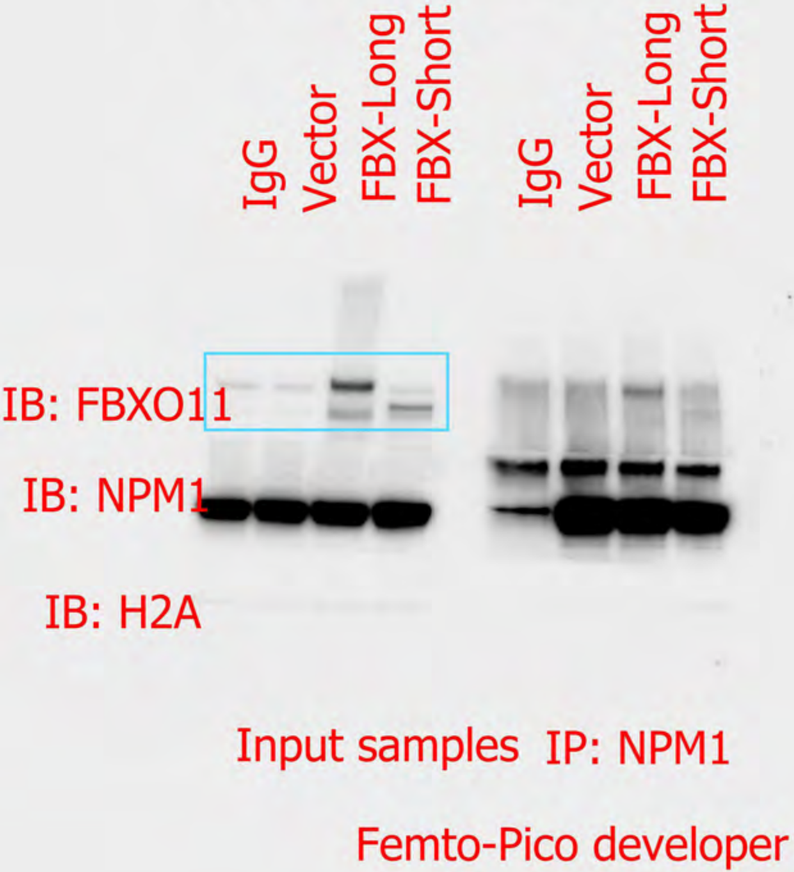

Unedited blot for Figure 2C - NPM1, input

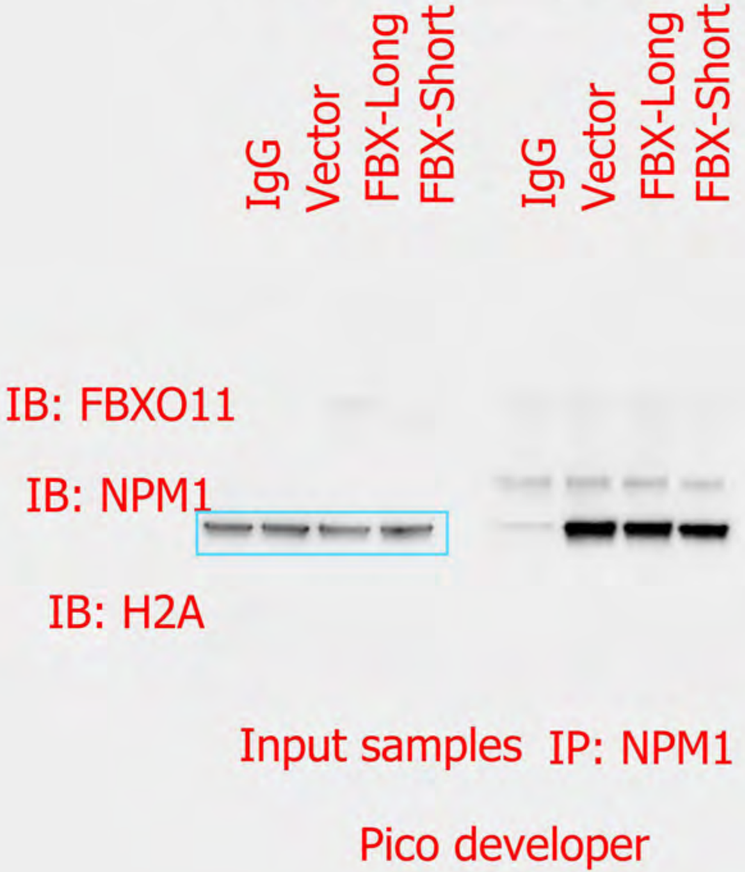

Unedited blot for Figure 2C - H2A, input

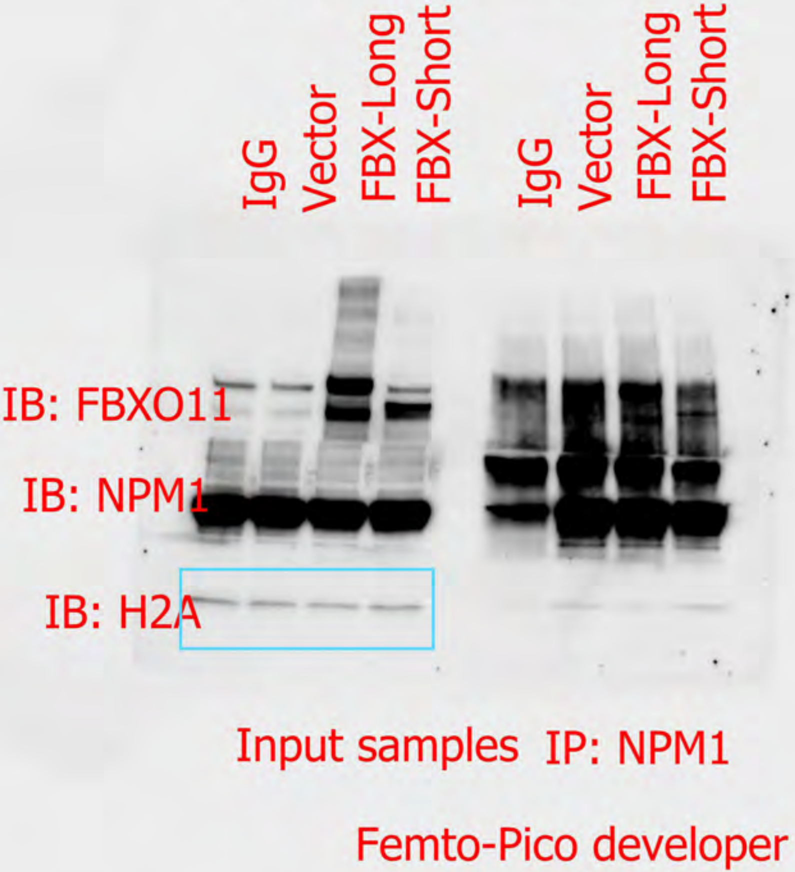

Unedited blot for Figure 2C - GFP-Ubiquitin, input

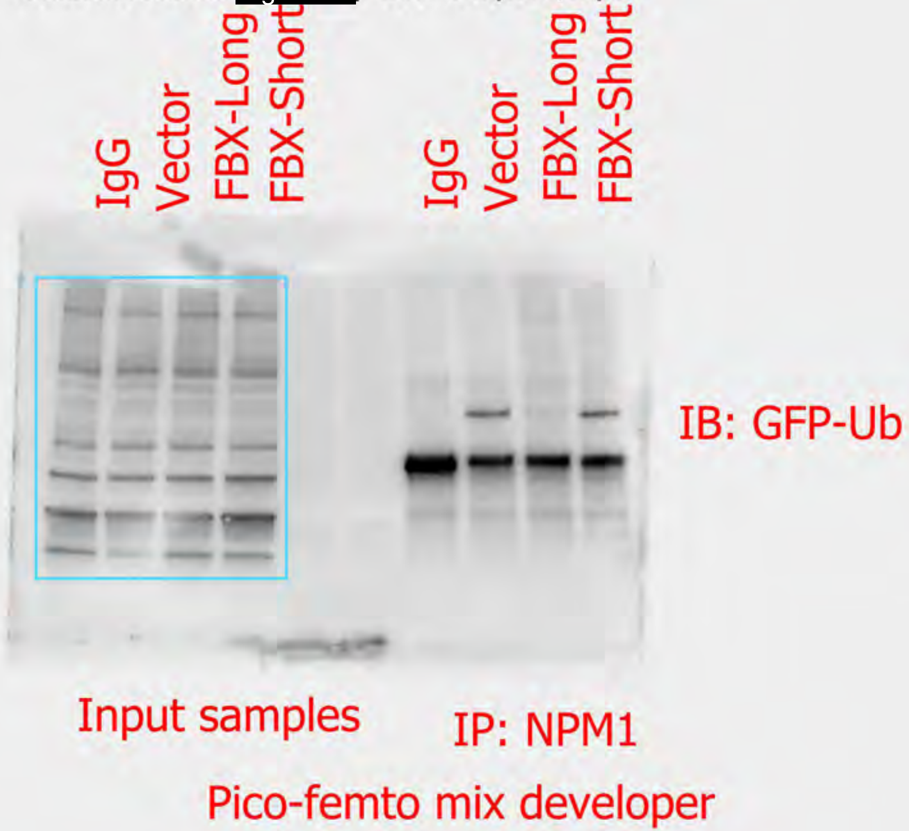

Unedited blot for Figure 2C - GFP-ubiquitin, IP

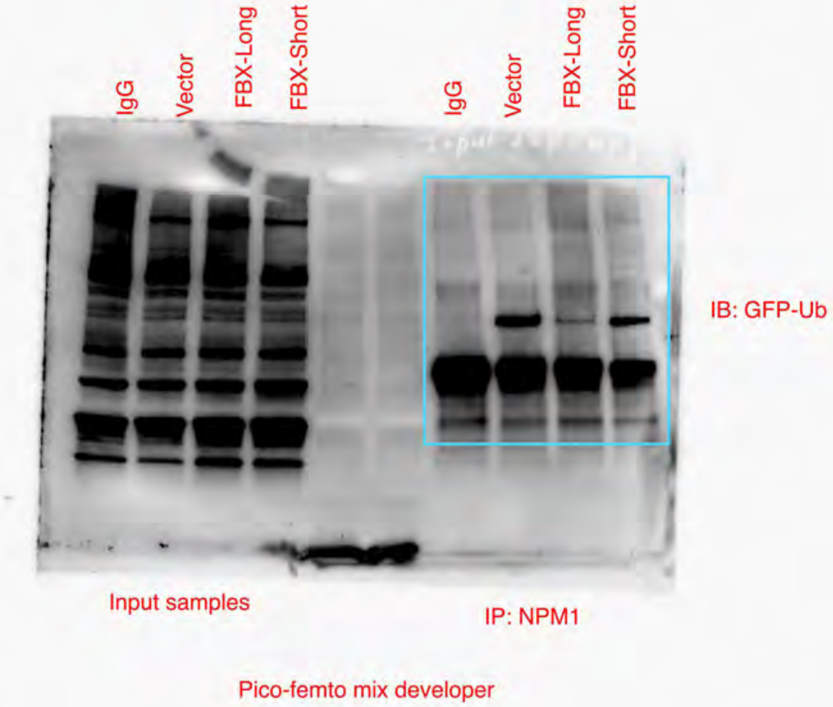

Unedited blot for Figure 2C - NPM1, IP

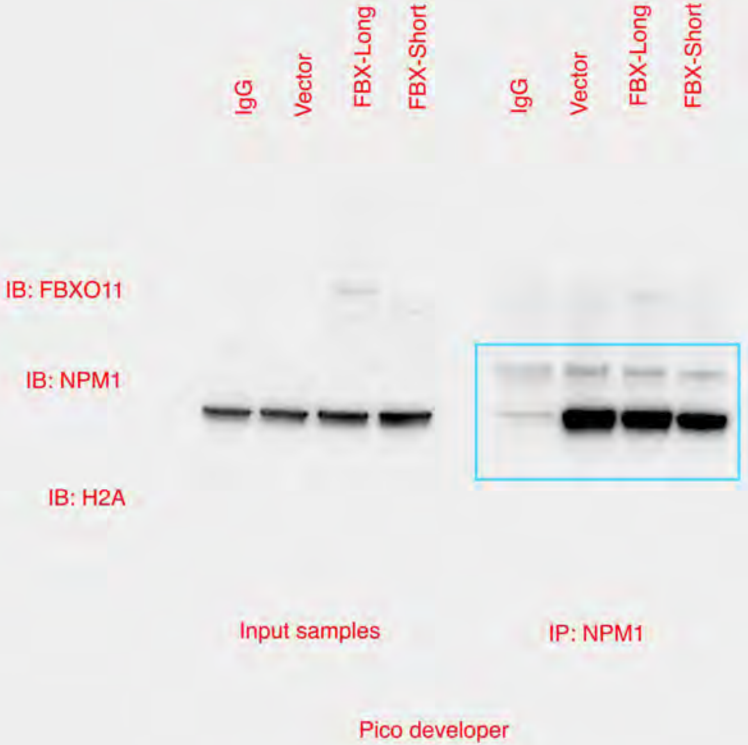

Unedited blot for Figure 3B - FBXO11

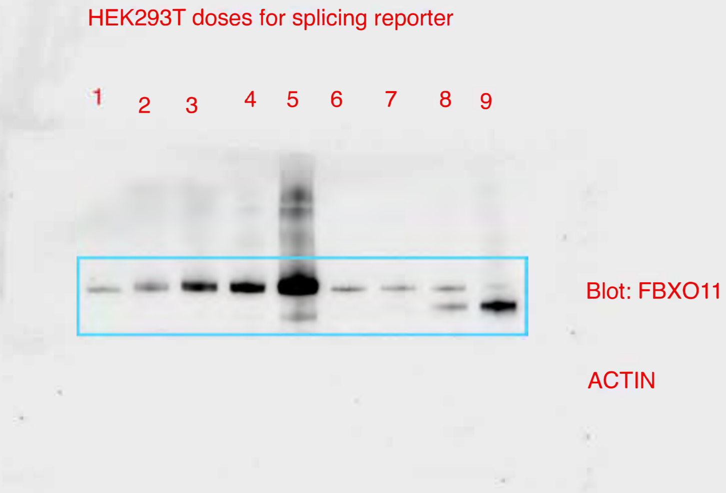

1. pcDNA
2. 0.5ug FBXO11 Long
3. 1.0ug FBXO11 Long
4. 2.0ug FBXO11 Long
5. 5.0ug FBXO11 Long
6. 0.5ug FBXO11 Short
7. 1.0ug FBXO11 Short
8. 2.0ug FBXO11 Short
9. 5.0ug FBXO11 Short

Unedited blot for Figure 3B - SYNCRIP

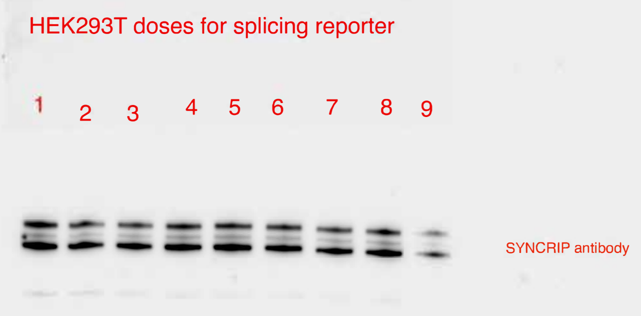

1. pcDNA
2. 0.5ug FBXO11 Long
3. 1.0ug FBXO11 Long
4. 2.0ug FBXO11 Long
5. 5.0ug FBXO11 Long
6. 0.5ug FBXO11 Short
7. 1.0ug FBXO11 Short
8. 2.0ug FBXO11 Short
9. 5.0ug FBXO11 Short

Unedited blot for [redacted] - FBXO11

Figure 6A

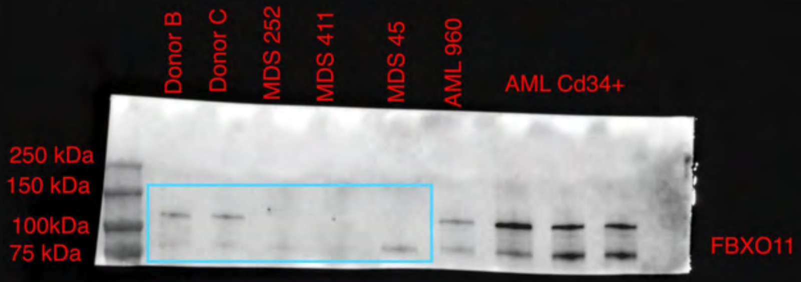

Unedited blot for Figure 6A - NPM1, H3

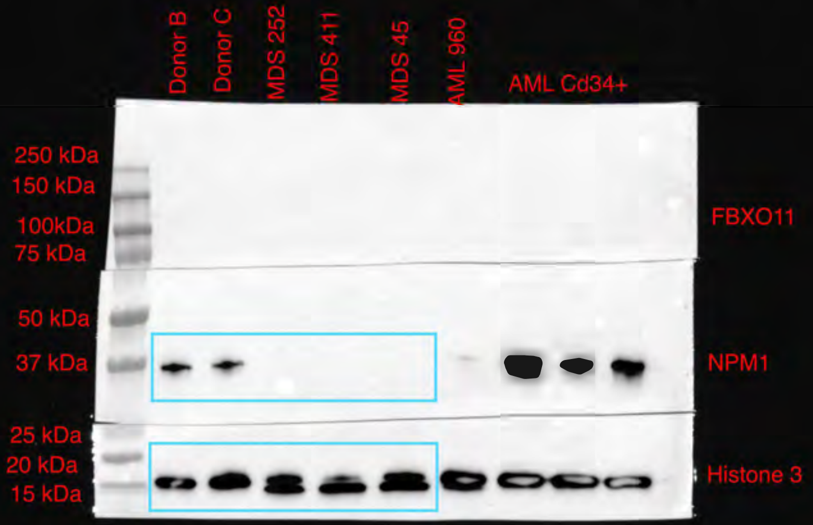

Unedited blot for [redacted] - FBXO11 set 2

Figure 6A

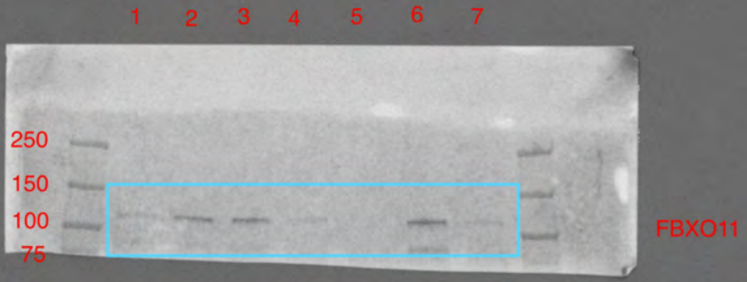

1. Healthy donor B
2. Healthy donor C
3. Healthy Donor E
4. MDS B
5. MDS C
6. MDS D
7. MDS G

Unedited blot for Figure 6A - NPM1 set 2

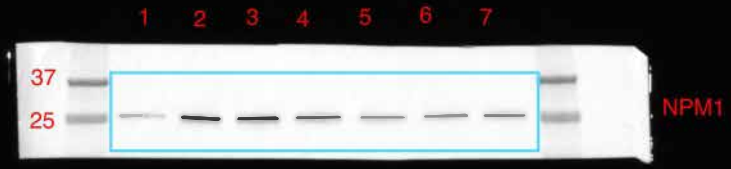

1. Healthy donor B
2. Healthy donor C
3. Healthy Donor E
4. MDS B
5. MDS C
6. MDS D
7. MDS G

Unedited blot for Figure 6A - H3 set 2

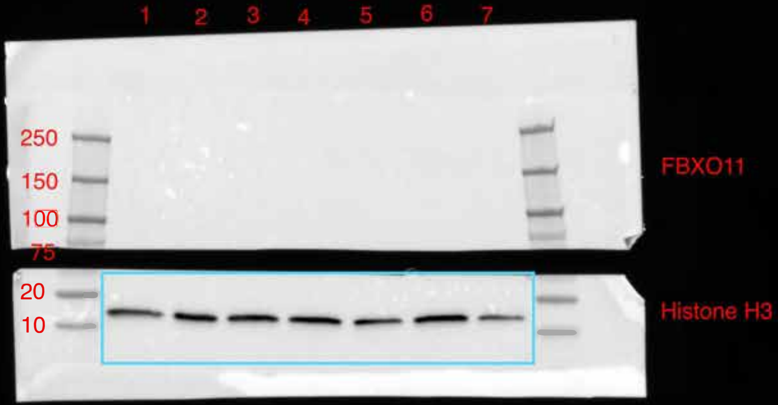

1. Healthy donor B
2. Healthy donor C
3. Healthy Donor E
4. MDS B
5. MDS C
6. MDS D
7. MDS G

Unedited blot for Figure 6O - FBXO11

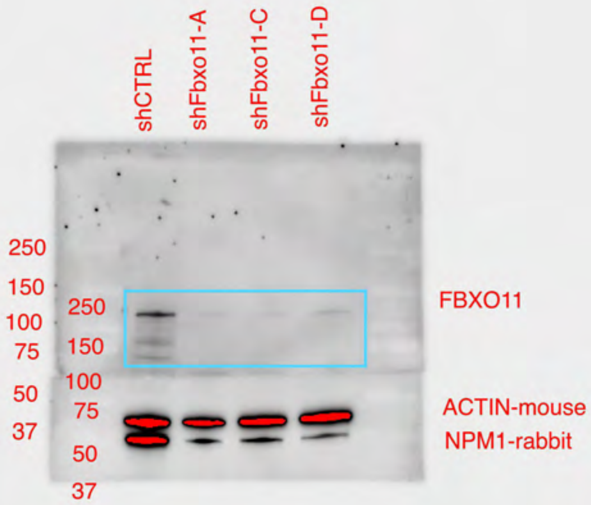

used 50-50 mix of pico and femto developer

Unedited blot for Figure 6O - ACTIN, NPM1

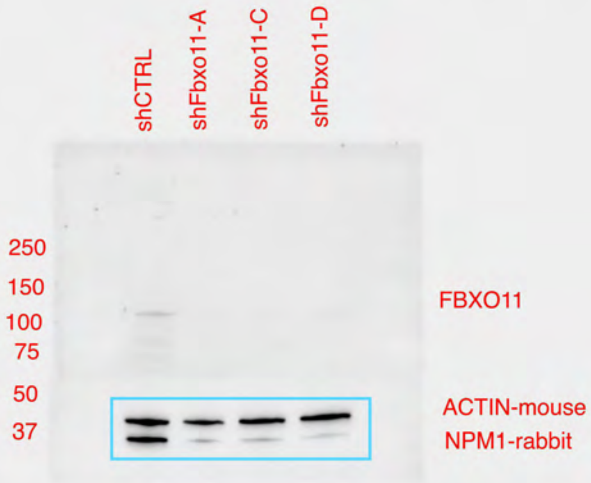

used 50-50 mix of pico and femto developer

Unedited blot for Figure S4A

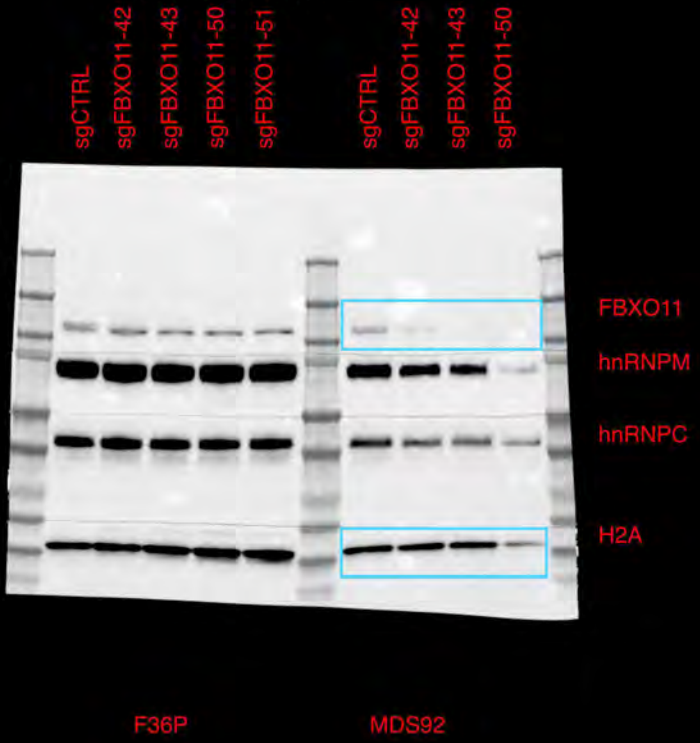

*not enough cells in sg51 for MDS groups

Unedited blot for Supp Figure 4B - FBXO11, ACTIN

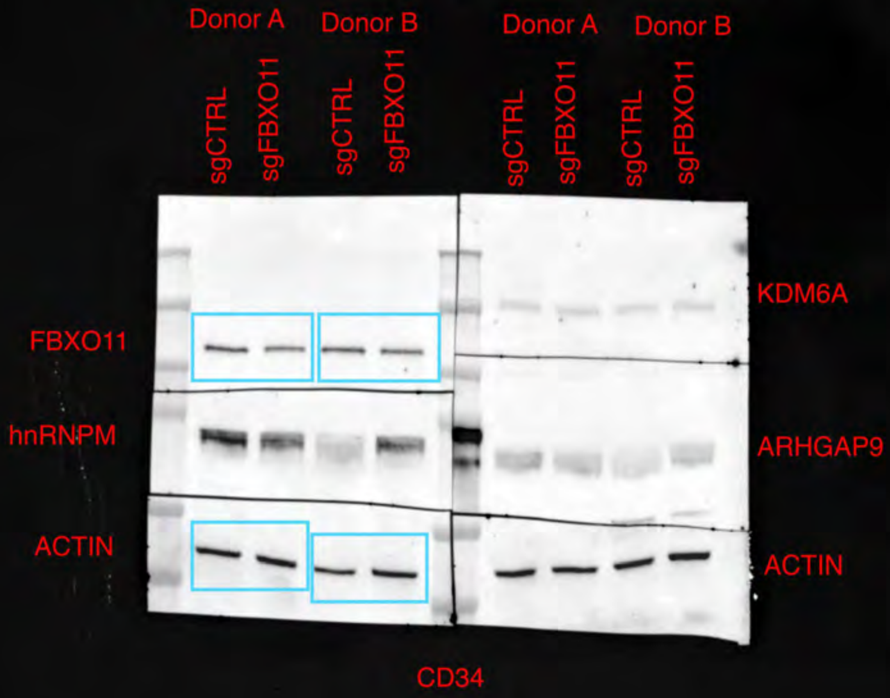

Unedited blot for Supp Figure [redacted] - FBXO11

5B

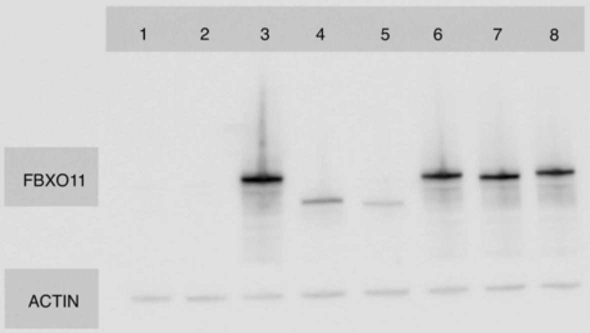

1. Negative CTRL
2. pcDNA only
3. FBXO11-Long
4. FBXO11-Short
5. FBXO11-Short delF-Box
6. FBXO11-P49Q
7. FBXO11-P45-Q53del
8. FBXO11-Q55-Q56 dup

Unedited blot for Supp Figure [redacted] - ACTIN

5B

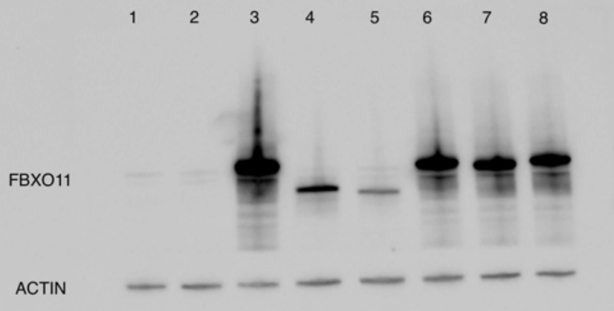

1. Negative CTRL
2. pcDNA only
3. FBXO11-Long
4. FBXO11-Short
5. FBXO11-Short delF-Box
6. FBXO11-P49Q
7. FBXO11-P45-Q53del
8. FBXO11-Q55-Q56 dup
